# Discovery of novel PDE9 inhibitors capable of inhibiting Aβ aggregation as potential candidates for the treatment of Alzheimer’s disease

**DOI:** 10.1038/srep21826

**Published:** 2016-02-25

**Authors:** Tao Su, Tianhua Zhang, Shishun Xie, Jun Yan, Yinuo Wu, Xingshu Li, Ling Huang, Hai-Bin Luo

**Affiliations:** 1 School of Pharmaceutical Sciences, Sun Yat-sen University, Guangzhou 510006, China

## Abstract

Recently, phosphodiesterase-9 (PDE9) inhibitors and biometal-chelators have received much attention as potential therapeutics for the treatment of Alzheimer’s disease (AD). Here, we designed, synthesized, and evaluated a novel series of PDE9 inhibitors with the ability to chelate metal ions. The bioassay results showed that most of these molecules strongly inhibited PDE9 activity. Compound **16** showed an IC_50_ of 34 nM against PDE9 and more than 55-fold selectivity against other PDEs. In addition, this compound displayed remarkable metal-chelating capacity and a considerable ability to halt copper redox cycling. Notably, in comparison to the reference compound clioquinol, it inhibited metal-induced Aβ_1-42_ aggregation more effectively and promoted greater disassembly of the highly structured Aβ fibrils generated through Cu^2+^-induced Aβ aggregation. These activities of **16**, together with its favorable blood-brain barrier permeability, suggest that **16** may be a promising compound for treatment of AD.

Alzheimer’s disease (AD), which is characterized by progressive cognitive decline, decline in language skills, and the presence of senile plaques, is a prevalent age-associated neurodegenerative disorder^1^. The etiology of AD remains elusive, but several characteristic pathological features, such as amyloid-β (Aβ) deposits^2^,^3^,^4^,^5^, low levels of acetylcholine^6^,^7^,^8^,^9^, inflammation, oxidative stress^10^,^11^,^12^, downregulation of the nitric oxide/soluble guanylyl cyclase (NO/sGC pathway)^13^,^14^, and impaired homeostasis of biometals^15^,^16^,^17^,^18^, might play significant roles in AD.

In the hippocampus and the cerebral cortex, the NO/sGC/cGMP signaling pathway plays a pivotal role in regulating synaptic transmission and plasticity, which are critical for learning and memory^13^,^14^. Recent studies have shown that the inhibition of the NO/sGC pathway alters the expression and activity of NOS, sGC, and phosphodiesterase (PDE) enzymes and contributes to Aβ neuropathology. Thus, PDE9 has been studied as a potential drug target for the treatment of Alzheimer’s disease^19^,^20^,^21^,^22^,^23^,^24^,^25^,^26^,^27^. Several new PDE9 inhibitors, including PF-04447943 and BI-409306, have been developed and tested for the treatment of AD in Phase II clinical trials^26^.

The impaired homeostasis of biometal is another important factor that contributes to the formation of Aβ oligomers, protofibrils, and amyloid fibrils^15^,^16^,^17^,^18^. It has been found that the concentration of metal ions in AD-affected brains is substantially higher than that in normal age-matched brains. When in excess, these metal ions, particularly Cu and Zn, bind Aβ peptides, promoting their aggregation. Several studies have found that metal ions modulate various pathways of Aβ aggregation and neurotoxicity as well the formation of reactive oxygen species (ROS) and oxidative stress^11^,^28^,^29^. Therefore, restoring the regulation of brain metal ion homeostasis has been thought to be a potentially efficient approach for the treatment of AD^15^,^30^,^31^,^32^,^33^,^34^. The typical examples of metal chelators are clioquinol (CQ) and its second generation derivative, PBT2, which have shown beneficial effects *in vivo* and have passed phase II clinical trials^35^.

Use of the multi-target-directed ligands (MTDLs) may be an appropriate and promising method to address the multifaceted nature of AD^5^,^10^,^36^,^37^,^38^,^39^,^40^,^41^,^42^. Here, we report the design, synthesis, and evaluation of a new series of multifunctional agents that combine the pharmacophores of both PDE9 inhibitors and biometal chelators (Fig. 1). Among them, **16**, with its favorable blood-brain barrier permeability, may be a promising compound for use in the treatment of AD.

## Results

### Chemistry

The synthesis of these compounds is illustrated in Fig. 2. First, the reaction of tetrahydro-4H-pyran-4-one or cyclopentanone with tert-butylcarbazate yielded imine, which was reduced with NaCNBH_3_. Then, the removal of the tert-butyloxycarboryl according to reported methods yielded the corresponding hydrazine hydrochloride (**3c** or **3d**)^26^,^27^. The reaction of compound **3** with 2-(ethoxymethylene)malononitrile yielded the pyrazoles (**4c** or **4d**), and the subsequent oxidation yielded the carboxamide (**5c** or **5d**). The reaction of the intermediate **5** with **2a** and **2b**, obtained by the methyl esterification of D-alanine and L-alanine and subsequent protection by the carbobenzoxy group, yielded compounds **6**, **7**, and **8**. Hydrogenation of the compounds **6–8** in the presence of Pd/C provided key intermediates **9–11**, which were then reacted with substituted salicylaldehyde or 2-pyridylaldehyde derivatives to produce the corresponding imine (**12–14**, **28**). The compounds **9–11** were reacted with the corresponding aldehydes and then reduced with NaBH_4_ to obtain other target compounds.

### Preliminary antioxidant experiments ***in vitro***

**Figure 1:**
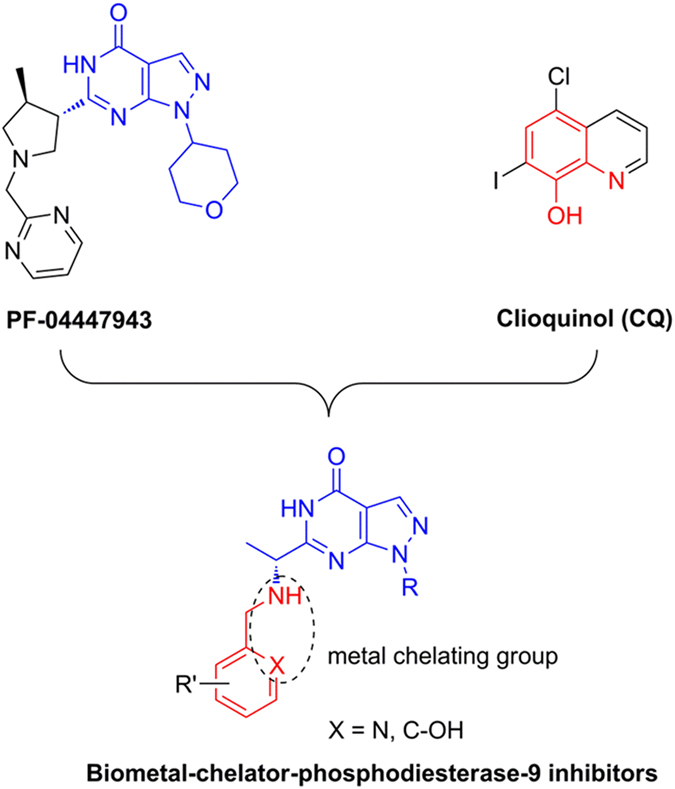
The design strategy of the multi-target-directed ligands.

**Figure 2:**
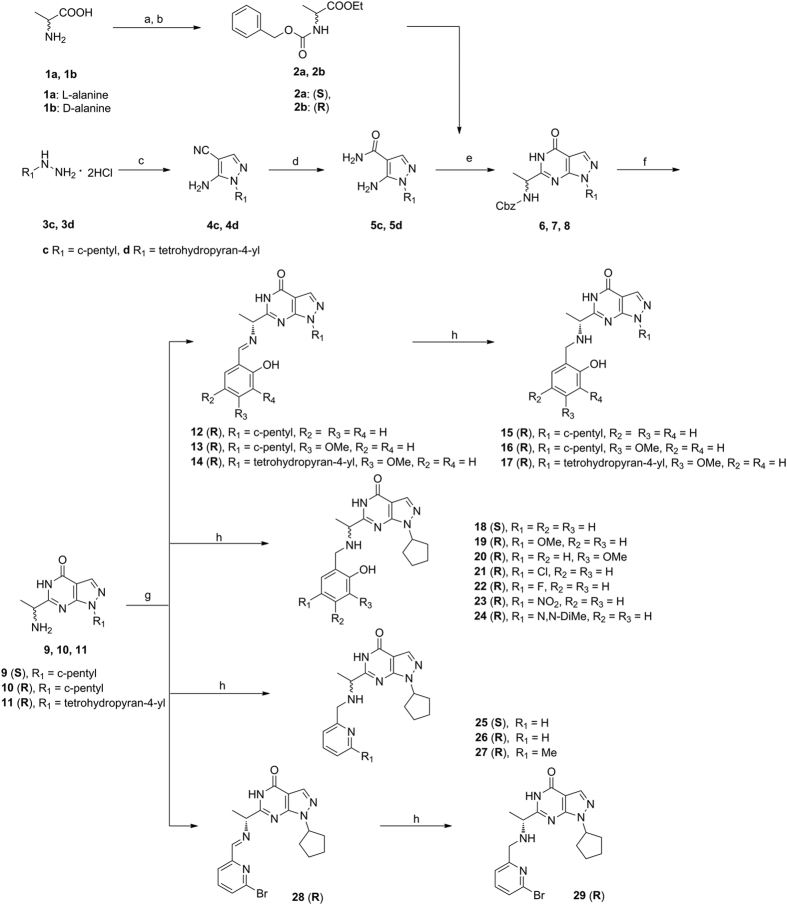
The synthesis of compounds 12–29. Reagents and conditions: (**a**) SOCl_2_, ethanol, reflux; (**b**) Benzyl chloroformate, Na_2_CO_3_, H_2_O, rt; (**c**) 2-(ethoxymethylene)malononitrile, Et_3_N, ethanol, rt to reflux; (**d**) 35% aq H_2_O_2_, aq ammonia, ethanol, rt; (**e**) **2a** or **2b**, NaH (80%), THF, rt; (**f**) Pd/C (10%), H_2_, MeOH; (**g**) substituted salicylaldehyde or substituted 2-pyridylaldehyde, MeOH, rt; (**h**) NaBH_4_, MeOH, rt.

Evidence of reactive oxygen species (ROS)-mediated injury has been observed in the AD brain, thus suggesting that antioxidants can be used as neuroprotective therapeutic agents in AD treatment^10^,^11^,^43^,^44^. Our preliminary experiments demonstrated that clioquinol is a weak antioxidant with an oxygen radical absorbance capacity of 0.62 ORAC-fluorescein (ORAC-FL) units, which was assessed using a vitamin E analog, Trolox, as a standard^36^,^37^. Therefore, we speculated that our target compounds, whose structures were optimized from clioquinol as illustrated in Fig. 1, may exert antioxidant effects. Additionally, melatonin, a well-known anti-oxidant, was also used as a reference compound.

As shown in Table 1, most of the compounds with an amine structure (for example, **15–22, 24**) showed excellent antioxidant properties as expected, with ORAC-FL values ranging from 3.50 to 5.84. These values were significantly better than that of the reference compound melatonin, which showed an ORAC-FL value of 2.00. Compounds **12–14**, with an imine structure in R_2_, showed low ORAC-FL values (less than 1.00 equivalent Trolox). However, **25–29**, which possessed pyridinyl (C ring) moiety, showed no antioxidant activities, owing to their electron-withdrawing properties.

### Inhibitory properties of PDE9 inhibitors

The PDE9 inhibitory activities of the compounds **12–29** are summarized in Table 1. Bay73-6691, which showed an IC_50_ of 48 nM, an inhibitory activity consistent with that reported in the literature (55 nM), was used as the positive control^20^. As indicated, most compounds were found to be potent PDE9 inhibitors with IC_50_ values less than 100 nM. Qualitative structure-activity relationship analyses showed that the inhibitory activity was influenced by the structures of R_2_. From a comparison of the potency of **12** (IC_50_ = 211 nM) with that of **15** (58 nM), and **28** (1160 nM) with that of **29** (38 nM), it appeared that the amine structures were more favorable than the corresponding imines for the inhibition of PDE9. However, this difference was negligible when methoxyl groups were present in the benzene ring of C (**13**, imine, 35 nM; **16**, amine, 34 nM). However, in the series of compounds containing an imine structure, the tetrahydropyran-4-yl (R_1_) appeared to be beneficial for the inhibitory activity. For example, compound **14**, in which the c-pentyl (R_1_) of **13** was replaced by tetrahydropyran-4-yl in the pyrazole moiety, showed the highest inhibitory effect, with an IC_50_ value of 12 nM. However, compound **16** (34 nM) exhibited superior inhibitory activity than did compound **17** (51 nM), indicating that the c-pentyl group was superior to the tetrohydropyran-4-yl in the amine series. Considering the relative stabilities of the imines and the amine *in vivo*, we focused on studying the effect of different R_2_ in the amine structures. Most compounds with different substituents on the moiety of C ring, such as **19–24**, **26–27**, and **29**, showed excellent PDE9 inhibitory activity, with IC_50_ values ranging from 32 nM to 59 nM. Compounds **16**, **19**, and **20**, with methoxyl groups in the C ring, and compounds **21–24**, **26–27**, and **29**, with different substituents in the C ring, exhibited similar IC_50_ values against PDE9.

Enantiopure drugs are very important in pharmaceuticals because different enantiomers of a chiral drug can bind different target receptors or enzymes. The results show that among the two pairs of the enantiomers, **15** vs **18** or **25** vs **26**, the (R)-configurations resulted in higher activities than the (S)-enantiomers. This result was consistent with earlier reports^20^,^26^,^27^.

### CoMFA statistical studies for PDE9 inhibitors

The comparative molecular field analysis (CoMFA)^43^,^44^ method was performed to determine the quantitative relationship between the structures and the IC_50_ values toward PDE9. The statistical parameters for the CoMFA models are shown in **SI 1** (Supplementary information). Based on the IC_50_ values, the CoMFA results generated a reasonable/acceptable model (*q*^2^ = 0.554 and *r*^2^ = 0.996) at optimal component six, which implied that the steric and electrostatic fields in this CoMFA model were sufficient to explain the inhibitory effects of the target compounds described in Table 1. The contour maps (**SI 1**), graphically converted from the resulting CoMFA model, can offer valuable insights into the intermolecular interactions between these compounds and their receptor, which may be helpful in the rational design of PDE9 inhibitors.

### Selectivity of inhibitors 16 and 22 across PDE families

Because PDEs are widely expressed in the central nervous system (CNS)^19^,^20^,^21^,^22^,^23^,^24^,^25^,^26^,^27^,^45^, and because they regulate a variety of physiological processes, achieving high PDE9 selectivity is critical for lowering the risk of side effects of the potential anti-AD lead compounds^35^,^42^. Considering the balance between the PDE9 inhibitory activity and the antioxidant capacity, we chose compounds **16** and **22**, which showed excellent performance in both assays, for subsequent evaluation of their specific affinity toward PDE families. The results, shown in Table 2, indicated that both **16** and **22** were highly selective PDE9 inhibitors against PDE1B (500-fold and 236-fold, respectively). The selectivity of **16** and **22** toward other PDEs, such as PDE2A3, PDE3A, PDE4D2, PDE5A1, PDE7A1, PDE8A1, and PDE10A2, was also evaluated. The results showed that they had good selectivity —50 ~ >1470-fold (Table 2). Because the selectivity of **16** toward all tested PDEs, except PDE10A2, was superior than that of **22**, **16** was chosen for further studies.

### Binding pattern of inhibitor 16 with PDE9

As assessed from the results of molecular docking experiments, inhibitor **16** showed a PDE9 binding pattern similar to that of our previously reported inhibitors **3r** (**SI 2**) and **28s**. Its pyrazolopyrimidinone ring formed two hydrogen bonds, 2.9 Å and 3.3 Å (relatively weak, not shown in **SI 2**), with the invariant Gln453 of PDE9 and was involved in aromatic π-stacking interactions with Phe456. These are two characteristic interactions of PDE9 inhibitors (**3r** and **28s**)^20^,^46^ with PDE9. Interestingly, the newly introduced amine N2 atom of **16** made a hydrogen bond, 3.0 Å with the side chain of the unique Tyr424 in PDE9, which may explain its 500-fold better PDE9 selectivity over PDE1 (Table 2). Similarly, in the crystal structure of PDE9 complexed with **28s**, Tyr424 formed a hydrogen bond with the amide oxygen of L-Ala of **28s**^20^, and changes to the nitrogen adjacent to D-Ala of **3r** have also been observed^46^.

As expected, compound **3r** showed a more negative docking score of −51.2 kcal/mol (CDOCKER-INTERACTION-ENERGY) than **16**, which showed a score of −47.7 kcal/mol. This result is in accordance with the inhibitory effects of the two compounds (0.6 nM and 34 nM).

### Lipid-water distribution coefficient and blood-brain barrier permeability *in vitro*

The 1-noctanol/water system was used to estimate the lipid–water distribution coefficient (logP) values^47^. These values were 1.10 and 1.50, respectively for compounds **16** and **22** (**SI 3** in Supplementary information).

Blood–brain barrier (BBB) permeability is another important feature of the drugs used in the treatment of CNS diseases. We measured the BBB permeability of **16** by using the parallel artificial membrane permeation assay^10^,^47^,^48^ of the blood–brain barrier (PAMPA-BBB). The permeability values of 13 selected commercial drugs were compared with reported values to validate the assay (**SI 4** in Supplementary information). The experimental data versus the reported values exhibited an excellent linear correlation:


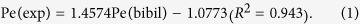


From the limit established by Di *et al.*^48^, we concluded that compounds with permeability values of above 4.7 × 10^−6^ cm s^−1^ (PBS-EtOH, 70 : 30) might cross the BBB (see Supplementary information). Compounds **16** and **22** were then tested with desipramine as the reference drug (**SI 4**). The permeability value of 17.03 obtained for **16** suggested that this compound might cross the BBB and exert multifunctional biological activities in the CNS.

### Metal-Chelating properties of 16

**Figure 3:**
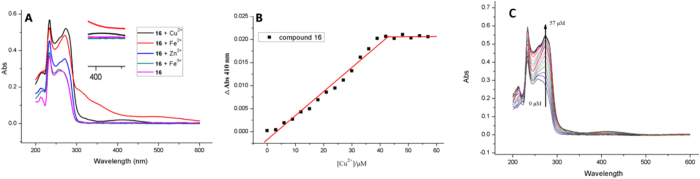
Metal-chelating properties of compound 16. (**A**) The UV spectrums of compound **16** (20 μM) alone and in the presence of CuSO_4_ (20 μM), FeSO_4_ (20 μM), Fe_2_(SO4)_3_ (20 μM), or ZnCl_2_ (20 μM) in 30% (v/v) ethanol/buffer (20 mM HEPES, 150 mM NaCl, pH = 7.4). (**B**) The determination of the stoichiometry of **16** (40 μM)-Cu^2+^ complex by molar ratio method. A breakpoint was observed at 1:1 ratio. The concentration of compound **16** was 40 μM. (**C**) UV-vis titration of compound **16** (40 μM) with Cu^2+^ in 30% (v/v) ethanol/buffer (20 mM HEPES, 150 mM NaCl, PH = 7.4). The concentration of Cu^2+^ was varied from 0 to 57 μM.

The ability of **16** to chelate bio-metals was studied by UV–vis spectrophotometry^31^,^49^,^50^. The spectral pattern of **16** with or without metal ions such as Cu^2+^, Fe^2+^, Fe^3+^, and Zn^2+^ are shown in Fig. 3. The pink line is the UV-vis spectrum of **16** between 200**–**600 nm; this spectrum showed two absorption peaks at 233 and 259 nm, respectively. After the incubation of **16** with Cu^2+^, the second peak shifted from 259 to 273 nm and a new peak appeared at 410 nm. Similar results were obtained upon incubation of **16** with Fe^2+^ or Zn^2+^. For example, the absorption peak shifted from 259 nm to 271 nm, and the optical intensity increased markedly after Fe^2+^ was added to the solution of **16**. These changes in absorbance indicated the formation of **16**-Metal ion (II) complex. The results of the UV-vis spectrophotometry assay showed that **16** failed to chelate Fe^3+^ effectively. However, this result requires further confirmation.

To evaluate the stoichiometry of the **16**–Cu^2+^ complex, a series of UV-vis spectrophotometry assays of **16** titrated against Cu^2+^ were performed. The final concentration of **16** was maintained at 40 μM, and the absorption spectra were recorded after different concentrations of Cu^2+^ were added. The stoichiometry of the 16-Cu2 ^+^ complex was evaluated by determining the changes in absorbance at 410 nm, where a new band had appeared (Fig. 3C). As shown in Fig. 3B, the absorption increased with an increase in Cu^2+^ concentration and reached a plateau at approximately 40 μM, which indicated that the stoichiometry of **16**–Cu^2+^ complex was 1:1.

### The ability of 16 to halt copper redox cycling via metal complexation

**Figure 4:**
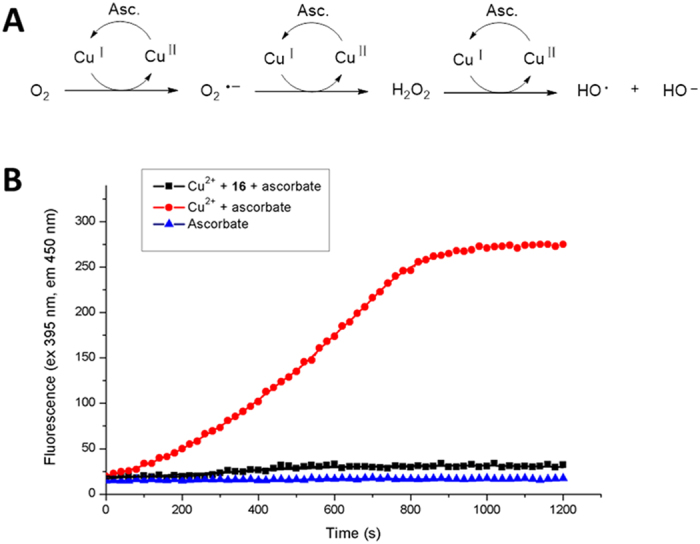
Fluorescence intensity of the Cu^2+^–ascorbate systems. (**A**) Redox cycling of copper in the presence of oxygen and ascorbate to produce OH·. (**B**) The fluorescence intensity of the Cu^2+^–ascorbate and Cu^2+^–**16**–ascorbate system; CCA (50 μM) and ascorbate (150 μM) were incubated in each system; [Cu^2+^] = 5 μM, [16] = 15 μM, in PBS (pH = 7.4).

Several bio-metals, especially the redox-active Cu^2+^, are involved in oxidative stress, which triggers neuronal cell death as seen in AD^31^,^51^. To evaluate the ability of the target compounds to halt the copper redox cycling via metal complexation under aerobic conditions, the Cu-ascorbate redox system was used as a model (Fig. 4)^31^,^52^. Fluorescent 7-hydroxyl-CCA, which was produced from coumarin-3-carboxylic acid (CCA) in the presence of hydroxyl radicals (OH·), was used to measure the reduction of hydroxyl radicals during the copper redox-cycling in the presence of ascorbate. As shown in Fig. 4B, the fluorescence intensity increased linearly for the first 12 min and then reached a plateau at 15 min. This process was fully inhibited when **16** was co-incubated with the Cu-ascorbate system, indicating that **16** had the capacity to halt the copper redox cycling by chelating the metal ions.

### Inhibition of Cu^2+^- induced Aβ_1-42_ aggregation

**Figure 5:**
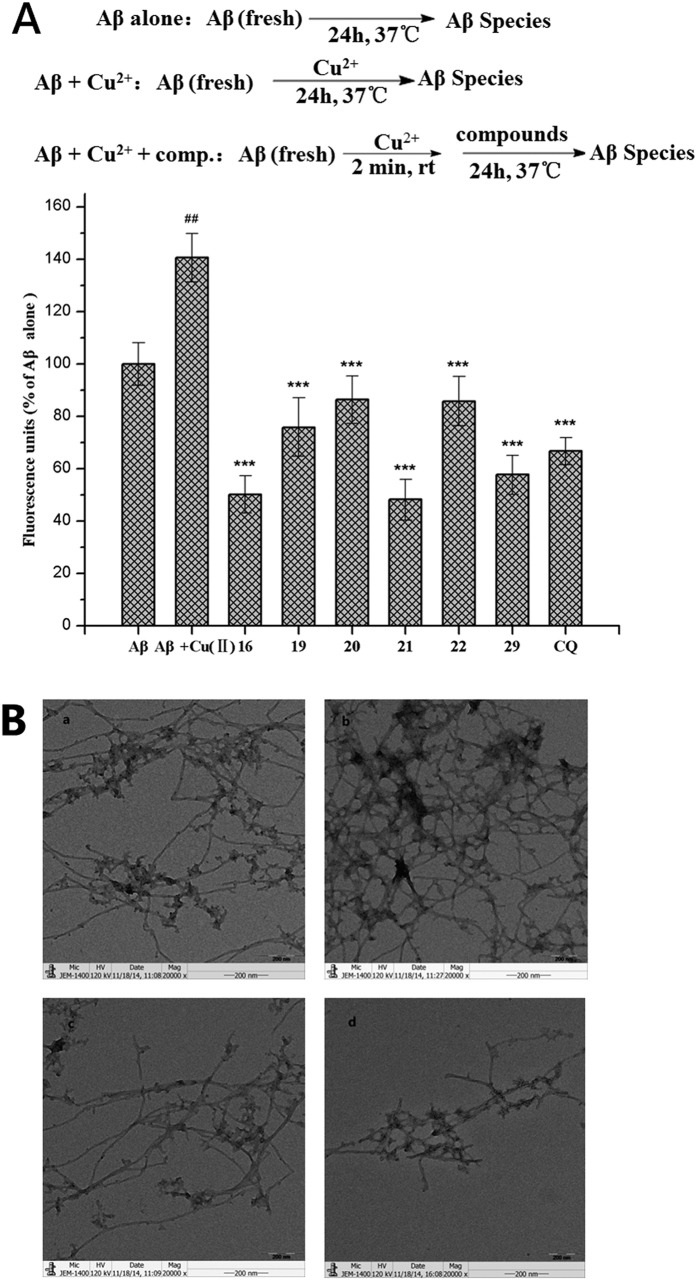
Inhibition of Cu^2+^- induced Aβ_1-42_ aggregation. (**A**) The results of the ThT binding assay. Statistical significance was analyzed by the Student’s *t*-test and ANOVA: (##)F_1,4_ = 32.94, p < 0.01, versus Aβ_1-42_ alone; (***) F_1,4_ = 138.03 (**16**), F_1,4_ = 67.95 (**19**), F_1,4_ = 60.50 (**20**), F_1,4_ = 158.06 (**21**), F_1,4_ = 62.59 (**22**), F_1,4_ = 116.09 (**29**) F_1,4_ = 106.69 (**CQ**), p < 0.001, versus Aβ_1-42_ + Cu^2+^ ([Aβ_1-42_] = 25 μM, [CQ] = 50 μM, [16] = [19] = [20] = [21] = [22] = [29] = 50 μM, [Cu^2+^] = 25 μM). (**B**) TEM images [[Aβ_1-42_] = 25 μM, [CQ] = 50 μM, [16] = 50 μM, [Cu^2+^] = 25 μM, 37 °C, 24 h]. (a) Aβ alone; (b) Aβ + Cu^2+^; (c) Aβ + Cu^2+^ + CQ; (d) Aβ + Cu^2+^ +  **16**.

To examine the effect of selected compounds on Cu^2+^- induced Aβ aggregation, we performed the thioflavin T (ThT) fluorescence and transmission electron microscopy (TEM) assay^5^,^30^,^37^,^53^,^54^ as shown in Fig. 5. As shown in Fig. 5A, a marked increase in the fluorescence was observed when Cu^2+^ was incubated with the Aβ for 24 h, indicating that Cu^2+^ accelerated the Aβ aggregation. The fluorescence significantly decreased when selected compounds and the known metal chelator CQ were incubated with Aβ in the presence of Cu^2+^ (inhibitory ratio: **16**, 64.7%; **21**, 65.7%; **29**, 59.1%; CQ, 52.5%, respectively), indicating that the tested compounds effectively inhibited the Cu^2+^-induced Aβ aggregation.

The result of the TEM analysis of the Aβ species was also consistent with that of ThT fluorescence assay. The TEM assay showed that the Cu^2+^-treated sample of fresh A*β* produced more fibrils than did the non-treated sample (Fig. 5a,b). When compound CQ or **16** and Cu^2+^ were incubated with A*β*, fewer A*β* fibrils were detected (Fig. 5c,d). When compound **16** was added to the samples, fewer fibrils were observed than in the presence of CQ.

### Disaggregation of Cu^2+^-induced Aβ_1-42_ aggregation fibrils

**Figure 6:**
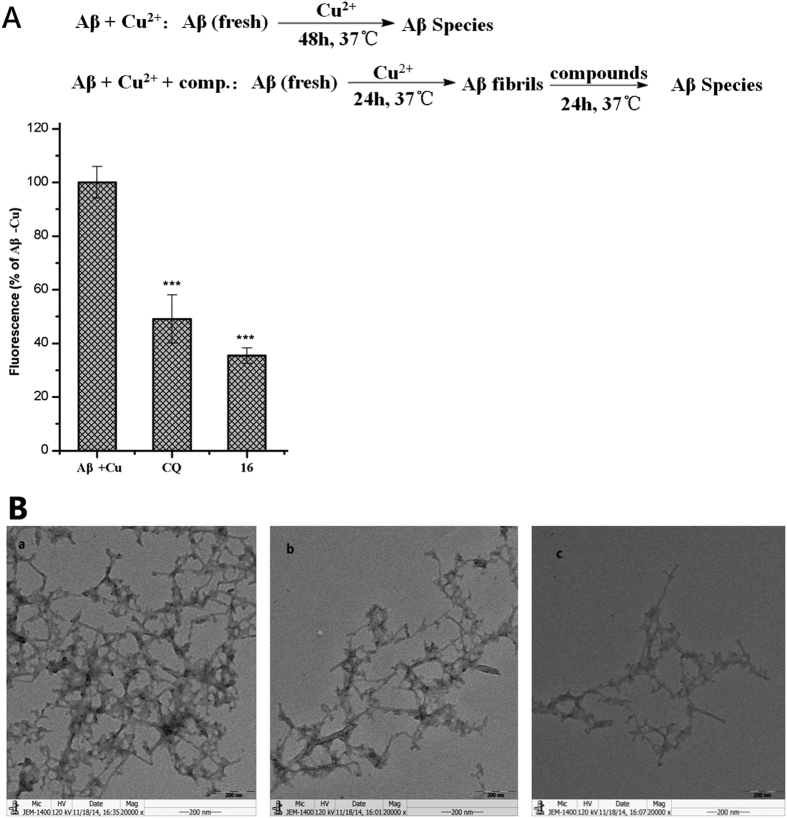
Disaggregation of Cu^2+^–induced Aβ_1-42_ aggregation fibrils. (**A**) ThT binding assay. Statistical significance was determined by Student’s *t*-test and ANOVA: (***)F_1,4_ = 68.02 (CQ), F_1,4_ = 110.17 (**16**), p < 0.001. ([Aβ_1-42_] = 25 μM, [CQ] = 50 μM, [16] = 50 μM, [Cu^2+^] = 25 μM) (**B**) TEM images [[Aβ_1-42_] = 25 μM, [CQ] = 50 μM, [16] = 50 μM, [Cu^2+^] = 25 μM, 37 °C, 24 h]. (a) Aβ + Cu^2+^, (b) Aβ + Cu^2+^ + CQ, (c) Aβ + Cu^2+^+**16**.

The ability of **16** to disaggregate the preformed Aβ_1-42_ fibrils was also studied (Fig. 6) by using reported methods^31^,^37^. First, fresh Aβ samples were incubated with Cu^2+^ at 37 °C for 24 h to obtain the Aβ fibrils. Then, **16** and CQ were added separately and incubated for an additional 24 h. The ThT binding assay showed that **16** and CQ markedly lowered the fluorescence intensity (**16**: 64.6% disaggregation; CQ: 50.9% disaggregation).

These results were also confirmed by the TEM assay. The incubation of Aβ_1-42_ in the presence of Cu^2+^ at 37 °C for 24 h produced well-defined Aβ fibrils (Fig. 6a). Notably, as assessed by TEM, incubation of the preformed fibrils with **16** or CQ for 24 h drastically reduced the amount of Aβ fibrils (Fig. 6Bc, **16**; Fig. 6Bb, CQ).

### Cell viability and intracellular antioxidant activity of 16

The antioxidant activity of **16** in SH-SY5Y cells was evaluated by using the cell-permeable dichlorofluorescein diacetate (DCFH-DA) as an indicator of ROS^29^,^37^,^45^. Trolox, a Vitamin E analog, was used as the positive control. First, the cytotoxicity of **16** toward the SH-SY5Y cells were determined by the colorimetric MTT assay. The results (**SI 5**) showed that **16** had nearly no toxicity below the 10 μM concentration. As shown in SI 6, the intracellular oxidative stress increased significantly after the treatment of SH-SY5Y cells with tert-butyl hydroperoxide, which resulted in the appearance of fluorescence (versus control). When the SH-SY5Y cells were incubated with tert-butyl hydroperoxide and the antioxidants (Trolox or **16**), the fluorescence intensities decreased by varying degrees, confirming their antioxidant activities. As shown in **SI 5**, **16** showed superior antioxidant activity to that of Trolox, even at relatively low concentrations (for example, 5 μM of **16** vs 10 μM of Trolox).

## Conclusion

In summary, a new series of multifunctional agents were designed and synthesized for the treatment of AD. These compounds combined the pharmacophores of PDE9 inhibitors and the bio-metal chelators. Among these compounds, **16** exhibited multivalent activities, such as an excellent inhibitory affinity of 34 nM towards PDE9, an antioxidant activity of 4.47 ORAC-FL units, significant inhibition of Cu^2+^-induced Aβ aggregation, and disaggregation of Aβ fibrils formed upon the treatment of Aβ with Cu^2+^. Moreover, our results showed that **16** is likely to cross the blood-brain barrier. All these properties suggest its potential as a compound for treatment of AD. Further investigations on candidate compounds are in progress.

## Methods

### General

All reagents used in reactions were obtained commercially and were used without further purification unless otherwise specified. Flash column chromatography was performed with silica gel (200–300 mesh) purchased from Qingdao Haiyang Chemical Co. Ltd. The mass spectra were recorded on an Agilent LC-MS 6120 instrument equipped with an ESI mass selective detector in positive ion mode. Melting points were determined on an SRS-Opti Melt automated melting point instrument. The NMR spectra were acquired on a Bruker Avance III spectrometer with TMS (Tetramethylsilane) as the internal standard. The purity (>95%) of the samples was determined by high-performance liquid chromatography (HPLC) with a TC-C_18_ column (4.6 × 250 mm, 5 μm) and acetonitrile/water as mobile phase at a flow rate of 1.0 mL/min.

### Oxygen Radical Absorbance Capacity (ORAC-FL) Assay

The experiments were performed as reported elsewhere^37^. The assays were performed in 75 mM PBS (pH 7.4) and all the tested compounds were dissolved in DMSO and diluted with the PBS. Trolox was used as a standard (1–10 μM final concentration). The tested compounds at different concentrations (20 μL, final concentration: 2 μM) and fluorescein (FL, 120 μL, final concentration: 300 nM) were placed in the wells of a black 96-well plate. The mixture was incubated for 10 min at 37 °C and then, APPH solution (60 μL, final concentration: 12 mM diluted by 75 mM PBS) was added rapidly. The plate was immediately transferred to a Spectrafluor Plus plate reader (Tecan Crailsheim, Germany), and the fluorescence was recorded every 60 s for 3 h with excitation at 485 nm and emission at 535 nm. A blank (FL + AAPH), with PBS replacing the compounds and Trolox was used for calibration in each assay. The compounds were tested at different concentrations (0.5–10 μM). The experiments were performed in three independent runs for each sample. The fluorescence measurements were normalized to that of the blank (without antioxidant). The area under the fluorescence decay curve (AUC) was calculated using the following equation (eq. 2):


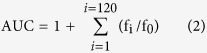


where *f*_0_ is the fluorescence reading at 0 min and *f*_i_ is the fluorescence reading at time i. The net AUC was calculated by the expression AUC_sample_ − AUC_blank_. Regression equations between net AUC and Trolox concentrations were calculated. The final results were expressed in μM of Trolox equivalent/μM of pure compounds.

### PDE activity assay

The assays were performed according to previously reported methods^20^,^46^. The enzymatic activities of the catalytic domains of PDE9 and other PDEs were measured by using cGMP or cAMP as substrates. The assay buffer contained 20–50 mM Tris–HCl (pH 7.5–8.0), 10 mM MgCl_2_ or 4 mM MnCl_2_, 1 mM DTT, and 10–20 nM ^3^H-cGMP or ^3^H-cAMP (20000–30000 cpm/assay, GE Healthcare). The reaction was performed at room temperature for 15 min and then terminated by the addition of 0.2 M ZnSO_4_. The addition of 0.25 M BaSO_4_ precipitated the generated ^3^H-GMP, whereas the unreacted ^3^H-cGMP remained in solution. The radioactivity in the supernatant was measured in 2.5 mL with an Ultim Gold liquid scintillation counter. The IC_50_ value of each inhibitor was measured at eight or more different concentrations of the inhibitor in the presence of ^3^H-cAMP or ^3^H-cGMP. Each measurement was repeated at least three times. The IC_50_ values were calculated by nonlinear regression method by using GraphPad Prism 5.0 Software.

### Docking methods

To identify the binding pattern of **16** with PDE9, the CDOCKER docking method of the Accelrys Discovery Studio 2.5.5 software was used. The crystal structure of the catalytic domain of human PDE9 complexed with **3r** (PDB code: 4QGE^46^) was used for the docking studies. The water molecules in the crystal structure were removed, except those coordinated with the two metal ions Zn^2+^ and Mg^2+^. Hydrogen atoms and charges were added to the receptor/ligand systems by using the CHARMm force field and the Momany-rone partial charge method, respectively. All ionizable residues in the systems were set to their protonation states at a neutral pH. The bound **3r** was used as a reference chemical to define the active site of PDE9. The radius of the input site sphere was set as 9 Å from the center of the binding site, and 50 random conformations were generated for each ligand. Other docking parameters were set to default values unless otherwise specified. Before the docking procedures, the bound ligand **3r** in 4QGE was redocked back to the same PDE9 enzyme, using different docking conditions and scoring parameters to assess the reliability of the CDOCKER method. In general, the docking may be considered successful if the RMSD (root mean square deviation) value of the optimum position is not more than a given threshold of 1.0 Å from the crystal structure after cluster analysis. As a result, the 25 positions of **3r** with the top docking scores had a mean of 0.92 Å for their RMSDs, which suggested that CDOCKER was suitable for use with the PDE9 system. Therefore, the same docking procedures were applied to **16** to generate its binding pattern with PDE9.

### Study of metal-chelating capacity

The experiments were performed according to previously reported methods^37^. The metal chelation was monitored spectrophotometrically using a UV–vis spectrophotometer. Typically, a solution of compound **16** (40 μM, final concentration) alone or **16** in the presence of CuSO_4_, FeSO_4_, Fe_2_(SO4)_3_, or ZnCl_2_ (40 μM, final concentration) in 30% (v/v) ethanol/buffer (20 mM HEPES, 150 mM NaCl, pH 7.4) was allowed to stand at room temperature for 30 min, and then the absorption spectrum was recorded at room temperature. The stoichiometry of the compound–Cu^2+^ complex was determined from molar ratio method as follows: compound **16** (40 μM, final concentration) was incubated with different concentration of CuSO_4_ (range from 0 to 57 μM), and the absorption spectra of the solutions were recorded after 30 min. The blank contained 30% (v/v) ethanol/buffer instead of Cu^2+^. The normalized absorbance of the newly formed absorption peak at 410 nm was plotted against the molar concentration of Cu^2+^. The breakpoint revealed the stoichiometry of the compound–Cu^2+^ complex.

### The determination of the lipid-water distribution coefficient

The distribution coefficients were determined by using the shake–flask method in 1-octanol/water system^47^. After shaking the tested compounds in 1-octanol/water (1:1) solution for 30 min, the distribution of the compounds in 1-octanol phase and water phase was determined by HPLC analysis. The lipid–water distribution coefficient was calculated according the following equation (eq. 3):


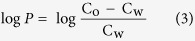


where C_o_ is the concentration of the test compound in water and C_w_ is its concentration in 1-octanol. The logP are mean values from at least three independent tests.

### *In Vitro* Blood-Brain Barrier Permeation Assay

The blood–brain barrier penetration capacity of the compounds was evaluated by using the parallel artificial membrane permeation assay (PAMPA). The drugs were purchased from Sigma and Alfa Aesar^10^,^47^,^48^. The porcine brain lipid (PBL) was obtained from Avanti Polar Lipids. The donor microplate (PVDF membrane, pore size of 0.45 nm) and the acceptor microplate were from Millipore. The acceptor 96-well microplate (COSTAR) was filled with 300 μL of a PBS/EtOH mixture (7:3) and the filter membrane was impregnated with 4 μL of PBL in dodecane (20 mg/mL). The compounds were dissolved in DMSO at a concentration of 5 mg/mL and diluted with the PBS/EtOH mixture (7:3) to a concentration of 100 μg /mL. Then, 200 μL of this solution was added to the donor wells and the wells were carefully placed on the acceptor plate, which was then incubated for 10 h at 25 °C in a vibrationless environment. After the incubation, the donor plate was removed and the concentration of compounds in the acceptor wells was determined with a UV plate reader (Flexstation 3). Each sample was analyzed at five wavelengths in four wells, and at least three independent runs were performed. The results are expressed as the mean ± standard deviation. In these experiments, 13 quality control standards of known BBB permeability were included to validate the analysis set. The *P*_e_ was calculated from the following equation (eq. 4) as reported by Faller *et al.* and Sugano *et al.*


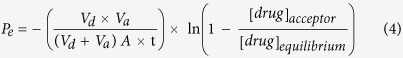


where V_d_ is the volume in the donor well, V_a_ is the volume in acceptor well, A is the filter area, t is the permeation time, [drug]_acceptor_ is the absorbance of the compound in the acceptor well, and [drug]_equilibrium_ is the theoretical equilibrium absorbance.

A plot of the experimental data versus the literature values yielded a linear correlation:


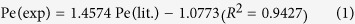


From this equation and the limit established by Di *et al.* Pe(lit.) = 4.0 × 10 −6 cm/s for blood − brain barrier permeation, we conclude that the compounds with permeability above 4.7 × 10^−6^ cm/s are able to cross the blood–brain barrier (Table 1 of the supporting information).

### Ascorbate Studies

The experiments were performed as reported previously^31^,^50^. With the exception of CuSO_4_ (dissolved in Milli-Q water) and **16** (dissolved in methanol and diluted in PBS), all agents were dissolved in phosphate buffer (PBS, 20 mM) containing NaCl (100 mM), pH 7.4, with a final sample volume of 200 μL. The following experimental procedure was used: the CCA (50 μM), compound **16** (15 μM), and copper (5 μM) were added successively into each well of a 96-well plate. Then, ascorbate (150 μM) was added to the solution, and the fluorescence was recorded every 20 s (λ_excitation_ = 395 nm, λ_emission_ = 450 nm). Each experiment was performed in triplicate. All test solutions contained 1 μM desferryl and 0.1% methanol.

### The ThT Assay

The experiments were performed as reported previously^37^. The Aβ_1−42_ sample (Sigma, US) was pretreated with hexafluoro-2-propanol (HFIP) and dissolved in ammonium hydroxide (1% v/v) to prepare a stock solution (2000 μM) that was aliquoted into small samples and stored at −80 °C. For assaying the inhibition of copper (II)-induced Aβ_1-42_ aggregation, the Aβ_1-42_ stock solution was diluted with HEPES buffer (20 μM HEPES, 150 μM NaCl, pH 6.6). Firstly, buffer (for Aβ anlone) or copper (II) (10 μL, 25 μM final concentration, for Aβ + Cu^2+^, drug treated groups) were added to the Aβ_1-42_ solution (10 μL, 25 μM final concentration and mixed for 2 min. Then, buffer or tested compounds (10 μL, 50 μM final concentration) were added to those solutions, which were then incubated at 37 °C for 24 h. Then, the sample was transferred to a black 96-well plate and diluted to a final volume of 200 μL with 50 mM glycine-NaOH buffer (pH 8.0) containing thioflavin-T (ThT, 5 μM). After incubation for 5 min in the dark, the fluorescence intensities were recorded (λex = 450 nm, λem = 485 nm). The percent inhibition of aggregation was calculated by the expression (1-IF_i_/IF_c_) ×100%, in which IF_i_ and IF_c_ are the fluorescence intensities obtained for Aβ in the presence and absence of inhibitors after subtracting the background, respectively.

For assaying the disaggregation of the copper (II)-induced Aβ_1-42_ fibrils, the Aβ_1-42_ stock solution was first diluted in HEPES buffer (20 μM HEPES, 150 μM NaCl, pH 6.6). The mixture of the peptide (10 μL, 25 μM final concentration) and copper (II) (10 μL, 25 μM final concentration) was then incubated at 37 °C for 24 h. Then, the test compound (10 μL, 50 μM final concentration) was added and incubated at 37 °C for an additional 24 h. Thereafter, 30 μL of the sample was diluted to a final volume of 200 μL with 50 mM glycine-NaOH buffer (pH 8.0) containing thioflavin-T (5 μM) in the dark. The detection method used was the same as that described above.

### Transmission Electron Microscopy (TEM) Assay

The assays were performed as reported previously^37^. For the TEM assay of copper (II)-induced Aβ_1-42_ aggregation and the disaggregation of the fibrils, the samples were pretreated as described for the ThT assay. Samples (10 μL) were placed on a carbon-coated copper/rhodium grid for 2 min. Then, each grid was stained with uranyl acetate (1%, 5 μL) for 2 min. After the excess staining solution was drained off, the specimen was transferred for imaging by a transmission electron microscope (JEOL JEM-1400).

### Cell Culture

Cell culture was performed as reported previously^37^. The human neuron-like cell line SH-SY5Y was obtained from the Institute of Biochemistry and Cell Biology, Shanghai Institute for Biological Sciences (Shanghai, China). The cells were cultured at 37 °C in a humidified atmosphere of 5% CO_2_ in Dulbecco’s modified Eagle’s medium (DMEM, GIBCO) supplemented with 10% fetal calf serum (FCS, GIBCO), 1 mM glutamine, 100 IU/mL penicillin, and 100 μg/mL streptomycin.

### Determination of Cytotoxicity

**C**ytotoxicity was determined as reported previously with the colorimetric MTT [3-(4,5-dimethyl-2-thiazolyl)-2,5-diphenyl-2Htetrazolium bromide] assay^37^. The SH-SY5Y cells were seeded at a density of 5 × 10^3^ cells/well in 96-well plates. After 24 h, the culture medium was replaced with medium containing the tested compound at different concentrations at 37 °C. After culturing for 48 h, 100 μL of medium containing 0.5 mg/mL MTT was added to each well. The cell were then incubated for 4 h at 37 °C in the dark. The solution was then gently aspirated from each well and the formazan crystals formed were dissolved with 100 μL of DMSO. The optical density of this solution was measured at 570 nm, and the cell viabilities were expressed as a percentage relative to the vehicle-treated control (0.5% DMSO was added to untreated cells).

### Antioxidant Activity in SH-SY5Y Cells

The antioxidant activity was determined as reported previously^37^. The SH-SY5Y cells were seeded at a density of 1 × 10^4^ cells/well in a 96-well plate. After 24 h, the culture medium was replaced with medium containing tested compounds, and the cells were cultured for an additional 24 h. Then, the cells were washed with PBS and incubated with 5 μM DCFH-DA (diluted by PBS) at 37 °C for 30 min. After discarding the solution and washing with PBS, the cells were treated with 0.1 mM t-BuOOH (a compound induce oxidative stress, diluted by PBS) for 30 min in the dark. Then, the fluorescence of the cells in each well was measured (λexcitation = 485 nm, λemission = 535 nm) with a multifunctional microplate reader (Flex Station 3). The antioxidant activity was expressed as a percentage relative to that of the control cells and calculated using the formula (F_t_ – F_nt_)/(F_t_’ – F_nt_) ×100, where F_t_ is the fluorescence value of the cells treated with the tested compound, F_t_’ is the fluorescence value of the cells not treated with the tested compound, and F_nt_ is the fluorescence value of the cells treated with t-BuOOH.

### Statistical Analysis

The experimental results are expressed as the mean ± standard deviation of at least three independent measurements. The data were subjected to Student’s *t*-test or one-way analysis of variance (ANOVA), followed by Dunnett’s test. p values ≤ 0.05 were considered statistically significant.

## Additional Information

**How to cite this article**: Su, T. *et al.* Discovery of novel PDE9 inhibitors capable of inhibiting Aβ aggregation as potential candidates for the treatment of Alzheimer's disease. *Sci. Rep.* **6**, 21826; doi: 10.1038/srep21826 (2016).

## Supplementary Material

Supplementary Information

## Figures and Tables

**Table 1:**
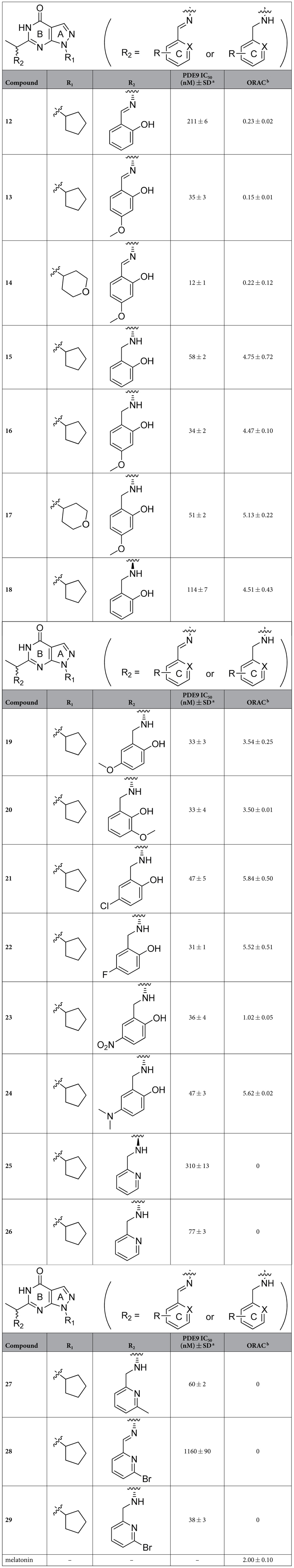
Inhibition towards PDE9 and antioxidant activities *in vitro*.

(**a**) The means ± SD of at least three independent experiments (**b**) the data are expressed as μmol of Trolox equiv/μmol tested compound. Trolox serves as a standard^36^,^37^. Melatonin also serves as a reference compound with the ORAC-FL value of 2.00.

**Table 2:** Inhibitory affinity of selected inhibitors with PDE families.

PDE catalytic domain	IC_50_(nM)								
	PDE9A2 (181–506)	PDE1B2 (10–487)	PDE2A3 (580–941)	PDE3A (679–1087)	PDE4D2 (86–413)	PDE5A1 (535–860)	PDE7A1 (130–482)	PDE8A1 (480–820)	PDE10A2 (448–789)
16	34 ± 2	(17 ± 4) × 10^3^ (494)	>50 × 10^3^ (1455)	>10 × 10^3^ (294)	(25 ± 2) × 10^3^ (739)	(1.9 ± 0.3) × 10^3^ (55)	>50 × 10^3^ (>1455)	>50 × 10^3^ (>1455)	(6.9 ± 0.1)×10^3^ (11567)
22	31 ± 1	(7 ± 3) × 10^3^ (234)	(13 ± 1) ×10^3^ (421)	>10 × 10^3^ (323)	(11 ± 2) × 10^3^ (355)	(1.6 ± 0.1) × 10^3^ (50)	>40 × 10^3^ (>1278)	>50 × 10^3^ (>1598)	(33 ± 1)×10^3^ (11100)

The numbers in parentheses are the fold of selectivity of PDE9 over other PDEs.
