# Supplementary material for: Discovery of novel PDE9 inhibitors capable of inhibiting Aβ aggregation as potential candidates for the treatment of Alzheimer’s disease
Source: Sci Rep. 2016 Feb 25;6:21826. doi: 10.1038/srep21826 (PMC4766439; doi:10.1038/srep21826)
Supplement: Supplementary Information [file srep21826-s1.pdf]

Supplementary information for

Discovery of novel PDE9 inhibitors that are capable of inhibiting Aβ aggregation as potential candidates for treatment of Alzheimer's disease

Tao Su,[+] Tianhua Zhang,[+] Shishun Xie, Jun Yan, Yinuo Wu, Xingshu Li, Ling Huang, * and Hai-Bin Luo*

School of Pharmaceutical Sciences, Sun Yat-sen University, Guangzhou 510006, China

* Hai-Bin Luo, Fax: +86-20-39943000, E-mail: luohb77@mail.sysu.edu.cn. * Ling Huang, Fax: +086-20-3994-3000, E-mail: huangl72@mail.sysu.edu.cn.

Content

SI 1. CoMFA statistical methods and results for PDE9 inhibitors

SI 2. Comparison on the binding of two PDE9 inhibitors 16 and 3r

SI 3. The logP values and permeability affinities from the PAMPA-BBB assay for selected compounds with its predicted penetration into the CNS

SI 4. In vitro blood-brain barrier permeation assay

SI 5. The antioxidant activity of 16 in SH-SY5Y cells

SI 6. The synthesis method and characterization of compounds 12- 29

SI 1. CoMFA statistical methods and results for PDE9 inhibitors

In CoMFA, the steric (S) and electrostatic (E) molecular fields are used. The starting models of all compounds were prepared and optimized using the standard Tripos force field with the software Tripos Sybyl 7.3.5. Their relevant partial-atomic charges were adopted from the empirical Gasteiger-Hückel (GH) set. Compound **16** served as an alignment template for the superposition. According to the standard CoMFA procedure, each compound was mapped onto a 3D lattice (20 Å × 20 Å × 20 Å) with grid points 2.0 Å apart. An sp3-hybridized carbon atom with a charge of +1 was employed as the interaction probe with the cut-off interaction energies set to 30 kcal/mol. The internal partial-least-squares (PLS) statistical analysis followed by the leave-one-out cross-validation was used to determine the number of optimal components and derive the CoMFA models by means of the standard implementation in the Sybyl package.

Table S1. Summary of the partial-least-squares statistical analyses on the training set based on the inhibitory data (−log IC_{50}) against PDE9

Model	q^2	*PC*	r^2	*S*	Relative contributions to the models (%)	
					S	E
CoMFA	0.216	6	0.992	0.050	41.6	58.4
CoMFA-focusing	0.554	6	0.996	0.035	32.3	67.7

S: steric, E: electrostatic. *r*, *q*, *S*, and PC are the cross-validated regression coefficient, conventional regression coefficient, standard deviation of estimate, and optimal number of principal components, respectively.

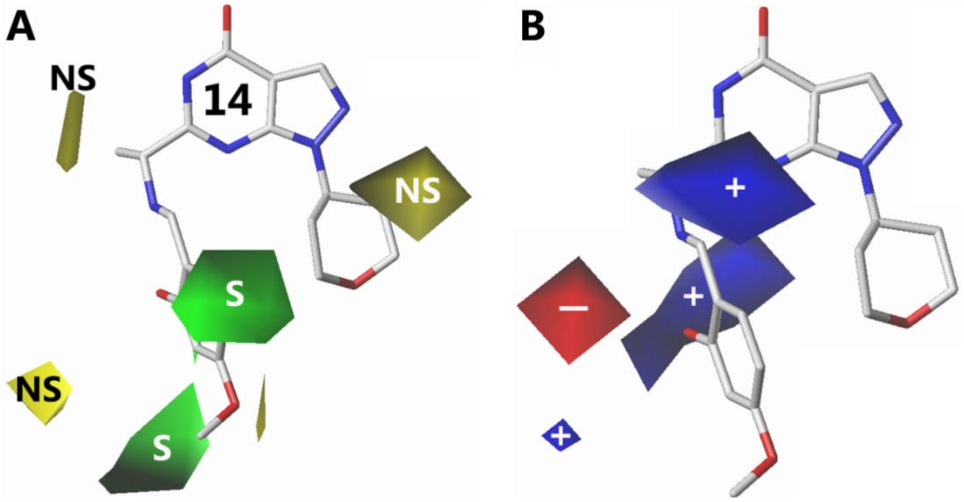

Figure S1. Contour maps of the CoMFA-focusing model with the steric (a) and electrostatic (b) fields based on the inhibitory data against PDE9 in the training set. Herein, compound **14** serves a reference. In (a), green contours (labeled as "**S**") emphasize areas that bulky substituents are favorable (w.r.t. stronger inhibition), whereas yellow contours ("**NS**") highlight regions that bulky groups are unfavorable. In (b), blue ("+") and red ("−") contours represent areas where electropositive and electronegative substituents in these positions will enhance the inhibition of compounds, respectively.

SI 2. Comparison on the binding of two PDE9 inhibitors 16 and 3r. (A) The binding pattern of **16** with PDE9 after molecular docking. PDE9 is presented in the surface mode. (B) Superposition between the crystal pose of PDE9-**3r** (yellow sticks, **3r** is our previously reported PDE9 inhibitor[46]) and the docking pose of PDE9-**16** (cyan).

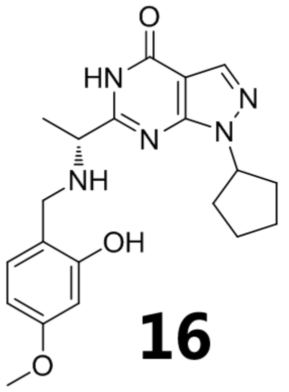

16

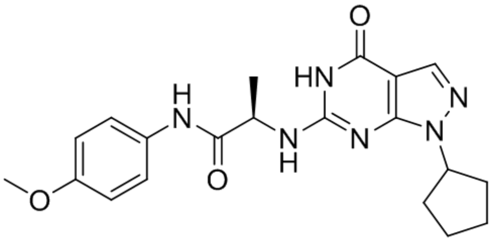

3r

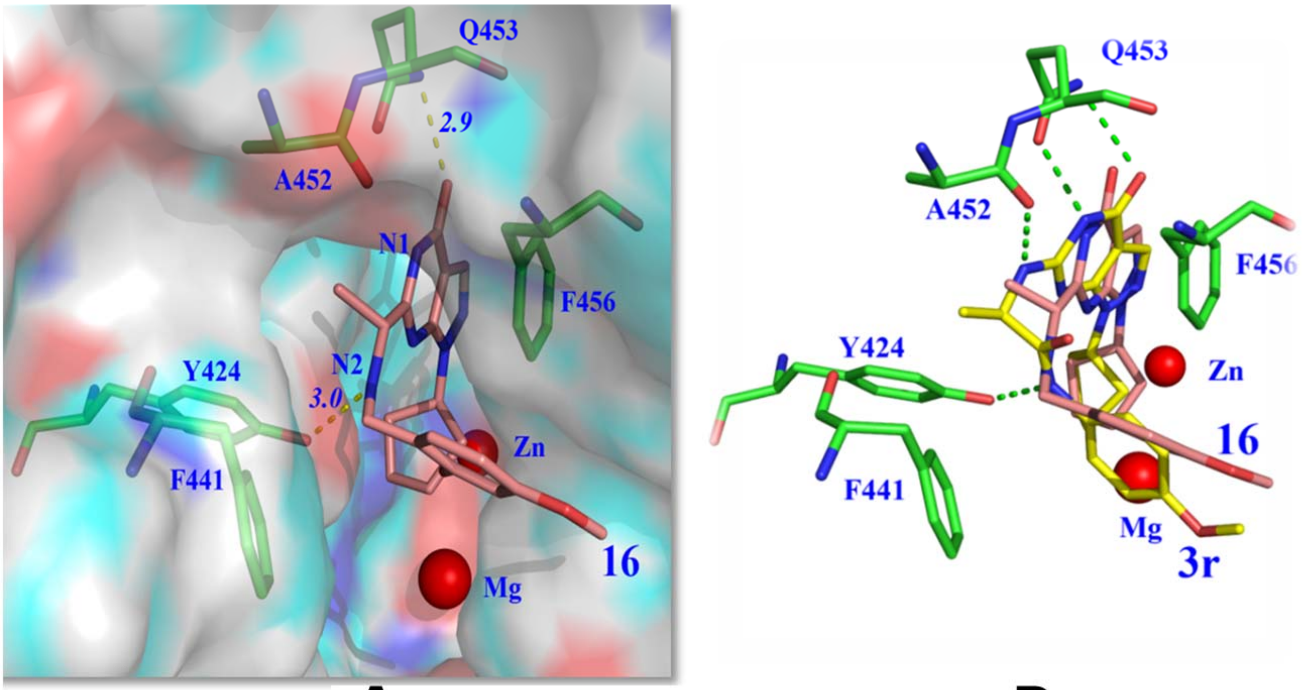

A **B**

SI 3. The logP values and permeability affinities (Pe 10^{-6} cm s^{-1}) from the PAMPA-BBB assay for selected compounds with its predicted penetration into the CNS. [a] The logP values are the means of at least three independent tests. [b] Values are expressed as the mean ± SD of three independent experiments. [c] not test.

compound	logP	Permeability (Pe 10^{-6} cm s^{-1}) [b]	prediction
**16**	1.10	17.03 ± 0.85	CNS+
**17**	--[c]	6.54 ± 0.76	CNS+
**22**	1.51	33.2 ± 4.76	CNS+
**desipramine**	--	16.4 ± 1.2	CNS+

SI 4. In Vitro Blood-Brain Barrier Permeation Assay

Permeability ($Pe \times 10^{-6}$ cm s^{-1}) in the PAMPA-BBB assay for 13 commercial drugs, used in the experiment validation.

Commercial drugs	Bibl	PBS:EtOH(70 :30)
Testosterone	17	22.3±1.4
Verapamil	16	21.2±1.9
Desipramine	12	16.4±1.2
Progesterone	9.3	17.7±1.2
Promazine	8.8	14.3±0.5
Chlorpromazine	6.5	6.0±0.3
Clonidine	5.3	5.1±0.3
Piroxicam	2.5	0.24±0.01
Hydrocortisone	1.9	0.65±0.01
Lomefloxacin	1.1	0.37±0.02
Atenolol	0.8	0.78±0.02
ofloxacin	0.8	0.37±0.02
theophylline	0.1	0.26±0.01

[a] Taken from reference 1. [b] data are the mean ± SD of three independent experiments

Lineal correlation between experimental and reported permeability of commercial drugs using the PAMPA-BBB assay Pe (exp.) = 1.4547Pe(bibl.)-1.0773 (R^2 = 0.9427).

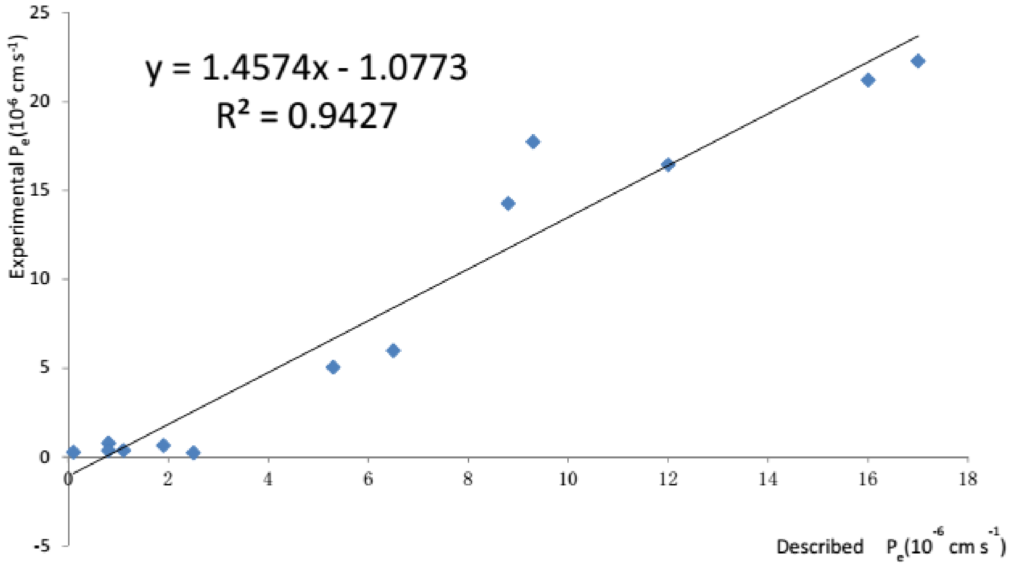

Ranges of Permeability of PAMA-BBB assays (Pe, 10^{-6} cm s^{-1})

Compounds of high BBB permeation (CNS+)	Pe > 4.7
Compounds of uncertain BBB permeation (CNS+/-)	4.7 > Pe > 1.8
Compounds of low BBB permeation (CNS-)	Pe < 1.8

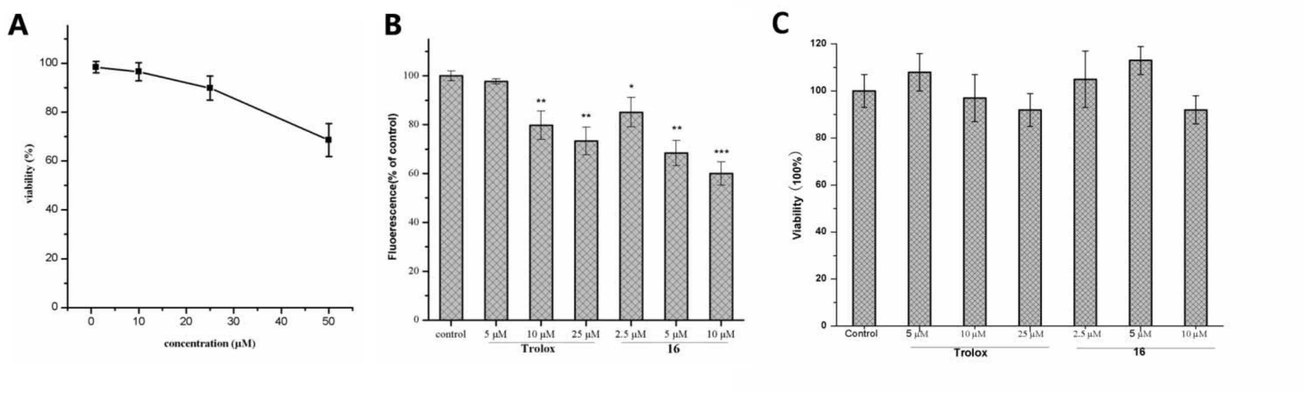

SI 5 The antioxidant activity of 16 in SH-SY5Y cells. (A) The cell viability of SH–SY5Y cells. (B) Percentage increase in intracellular ROS induced by exposure to t-BuOOH, determined by DCFH-DA. Statistical significance was analyzed by using ANOVA: (*) $F_{1,4} = 9.83$, $p < 0.5$, versus control, (**) $F_{1,4} = 24.78$ (Trolox, 10 uM), $F_{1,4} = 30.82$ (Trolox, 25 uM), $F_{1,4} =54.95$ (**16**, 5 uM), $p < 0.01$, versus control, (***) $F_{1,4} = 107.92$, $p < 0.001$, versus control. (C) The cell viability of SH–SY5Y cells after treated with trolox and **16**.

SI 6. The synthesis method and characterization of compounds 12- 29.

(S)-ethyl 2-(((benzyloxy)carbonyl)amino)propanoate (2a). To a solution of L-alanine (8.91 g, 100 mmol) was in 120 mL of ethanol, thionyl chloride (17.845g, 150mmol) was added dropwise. After refluxed for 5h, the mixture was cooled to room temperature, and evaporated the solvent to obtain the crude (S)-ethyl-2-aminopropanoate hydrochloride as oil. After the crude product was dissolved in 150 mL of water and cooled to 0℃, sodium carbonate (211 g, 200mmol) was added portionwise in 15 min and the solution was stirred for another 15min. Benzyl chloroformate (17.0 g, 100mmol) was added to the solution, and then the mixture was warmed to room temperature. After stirred for 5 h, the mixture was extracted with ethyl acetate. The combined the organic layer was dried with anhydrous sodium sulfate and evaporated the solvent under vacuum to obtain **2a** as colorless oil (two steps, yield = 89%). ^{1}H NMR (400 MHz, $CDCl_3$) δ 7.40 – 7.34 (m, 5H), 5.31 (s, 1H), 5.11 (s, 2H), 4.41 – 4.32 (m, 1H), 4.20 (dd, J = 14.0, 7.0 Hz, 2H), 1.41 (d, J = 7.2 Hz, 3H), 1.27 (t, J = 6.8 Hz, 3H). ^{13}C NMR (101 MHz, $CDCl_3$) δ 173.04, 155.69, 136.39, 128.49, 128.10, 126.90, 66.83, 61.40, 49.70, 18.56, 14.09.

(R)-ethyl-2-(((benzyloxy)carbonyl)amino)propanoate(2b). D-alanine was used according the above procedure to yield **2b** as colorless oil (two steps, yield = 90%).^{1}H NMR (400 MHz, $CDCl_3$) δ 7.40 – 7.34 (m, 5H), 5.31 (s, 1H), 5.11 (s, 2H), 4.41 – 4.32 (m, 1H), 4.20 (dd, J = 14.0, 7.0 Hz, 2H), 1.41 (d, J = 7.2 Hz, 3H), 1.27 (t, J = 6.8 Hz, 3H). ^{13}C NMR (101 MHz, $CDCl_3$) δ 173.04, 155.69, 136.39, 128.49, 128.10, 126.90, 66.83, 61.40, 49.70, 18.56, 14.09.

5-amino-1-cyclopentyl-1*H*-pyrazole-4-carbonitrile (4c). A solution of cyclopentylhydrazine dihydrochloride (13.07 g, 0.75mol) in anhydrous ethanol (180 mL) was added triethylamine (26.54 g,

0.262mol) at 0 ℃. The resulting mixture was stirred for 1 h and then 2-(ethoxymethylene)malononitrile (9.15 g, 0.075 mol) in 100 mL of anhydrous ethanol was dropped slowly (to keep the reaction temperature below 5 ℃). The mixture was stirred at room temperature for 10 h, and then refluxed for 2 h. After removal of the solvent under vacuum, 250 mL of water was added to the residue to obtain an yellow solid, which was collected, washed with additional 150 mL of water and 150 mL of 1:1 hexane and ether, and dried to give 10.83 g yellow solid (yield = 82 %). ^{1}H NMR (400 MHz, DMSO) δ 8.11 (s, 1H), 5.51 (d, *J* = 6.8 Hz, 1H), 4.50 – 4.38 (m, 1H), 3.39 (s, 1H), 2.03 – 1.93 (m, 2H), 1.88 – 1.79 (m, 2H), 1.76 – 1.68 (m, 2H), 1.63 – 1.53 (m, 2H). ^{13}C NMR (101 MHz, DMSO) δ 157.33, 134.01, 114.78, 76.01, 62.18, 31.82, 23.64.

5-amino-1-(tetrahydro-2H-pyran-4-yl)-1*H*-pyrazole-4-carbonitrile (4d). A solution of (tetrahydro-2H-pyran-4-yl)hydrazine dihydrochloride (4.77 g, 0.025 mol) in anhydrous ethanol (60 mL) was added triethylamine (8.93g, 0.088 mol) at 0 ℃. The resulting mixture was stirred for 1 h and then 2-(ethoxymethylene)malononitrile (4.616 g, 0.037 mol) in 15 mL of anhydrous ethanol was dropped slowly (to keep the reaction temperature below 5 ℃). The mixture was stirred at room temperature for 10 h, and then refluxed for 2 h. After removal of the solvent under vacuum, 100 mL of water was added to the residue to obtain an yellow solid, which was collected, washed with additional 30 mL of water and 30 mL of 1:1 hexane and ether, and dried to give 4.324 g yellow solid (yield = 90 %). ^{1}H NMR (400 MHz, DMSO) δ 7.55 (s, 1H), 6.57 (s, 1H), 4.32 – 4.25 (m, 1H), 3.94 (dd, *J* = 11.1, 4.0 Hz, 2H), 3.41 – 3.36 (m, 3H), 1.94 – 1.83 (m, 2H), 1.72 – 1.69 (m, 2H). ^{13}C NMR (101 MHz, DMSO) δ 150.42, 139.90, 115.14, 72.10, 66.02, 51.82, 31.41.

5-amino-1-cyclopentyl-1*H*-pyrazole-4-carboxamide (5c). To the stirred solution of 5-amino-1-cyclopentyl-1H-pyrazole-4-carbonitrile **4c** (5.3 g, 30 mmol) in 40 mL ethanol, 30% aqueous H_2O_2 (4 mL) was added and then aqueous ammonia (12 mL). The reaction mixture was stirred at ambient temperature for 48 h and then quenched with saturated sodium thiosulfate solution (50 mL). The resulting solution was concentrated under reduced pressure to remove most of the ethanol. The solid generated was filtrated and washed with water (40 mL × 2), dried in vacuum to yield faint yellow solid (5.3 g, 91% yield). ^{1}H NMR (400 MHz, DMSO) δ 7.63 (s, 1H), 7.16 (br s, 1H), 6.62 (br s, 1H), 6.13 (m, 1H), 4.57 – 4.45 (m, 1H), 3.39 (s, 1H), 2.00 – 1.87 (m, 2H), 1.87 – 1.72 (m, 4H), 1.63 – 1.50 (m, 2H). ^{13}C NMR (101 MHz, DMSO) δ 165.73 (d, J = 0.8 Hz), 148.24 (t, *J* = 4.9 Hz), 136.27, 96.17 (d, *J* = 2.9 Hz), 55.30 (d, *J* = 1.5 Hz), 30.65, 23.48.

5-amino-1-(tetrahydro-2*H*-pyran-4-yl)-1*H*-pyrazole-4-carboxamide (5d). The intermediate **5d** was prepared analogous to the procedure of **5c** by using 5-amino-1-(tetrahydro-2H-pyran-4-yl)-1H-pyrazole-4-carbonitrile (**4d**) as the starting material (85% yield). ^{1}H NMR (400 MHz, DMSO) δ 7.65 (s, 1H), 7.19 (br s, 1H), 6.63 (br s, 1H), 6.22 – 6.19 (m, 1H), 4.31 – 4.19 (m, 1H), 3.96 – 3.92 (m, 2H), 3.42 – 3.38 (m, 3H), 1.97 – 1.87 (m, 2H), 1.72 – 1.68 (m, 2H). ^{13}C NMR (101 MHz, DMSO) δ 166.79, 148.94 (t, *J* = 4.9 Hz), 137.50, 97.17 (t, *J* = 2.5 Hz), 66.70, 51.75 (d, *J* = 1.6 Hz), 32.09.

(S)-benzyl-(1-(1-cyclopentyl-4-oxo-4,5-dihydro-1*H*-pyrazolo[3,4-d]pyrimidin-6-yl)ethyl)carbamate (6). A stirred solution 5-amino-1-cyclopentyl-1*H*-pyrazole-4-carboxamide **5c** (2.91g, 15 mmol) in anhydrous THF was treated NaH (2.70 g, 90 mmol, 80% dispersion in mineral oil) at 0 ℃，the mixture was stirred for additional 15 min. The (S)-ethyl 2-(((benzyloxy)carbonyl)amino)propanoate

2a (16.96 g, 67.5 mmol) was added dropwise in 30 min and then stirred in room temperature for 14 h. After removal of the solvent, the residue was added 150 mL of water and extracted with ethyl acetate (80 mL × 3). The combined organic phase was dried (Na_2SO_4), evaporated the solvents under vacuum to afford crude product, which was purified by flash column chromatography (dichloromethane / methanol = 150 / 1) to give pure **6** (1.76 g, yield = 31%). ^{1}H NMR (400 MHz, $CDCl_3$) δ 11.90 (s, 1H), 8.05 (s, 1H), 7.32 – 7.28 (m, 5H), 5.98 (d, *J* = 6.1 Hz, 1H), 5.23 – 5.05 (m, 3H), 4.98 – 4.83 (m, 1H), 2.10 (dd, *J* = 13.6, 6.9 Hz, 4H), 2.02 – 1.92 (m, 2H), 1.79 – 1.69 (m, 2H), 1.60 (d, *J* = 7.1 Hz, 3H). δ 160.26, 159.79, 155.83, 151.90, 136.12, 134.56, 128.50, 128.18 (d, *J* = 7.5 Hz), 104.41, 67.18, 57.89, 50.43, 32.47, 24.78, 20.56.

(R)-benzyl-(1-(1-cyclopentyl-4-oxo-4,5-dihydro-1*H*-pyrazolo[3,4-d]pyrimidin-6-yl)ethyl)carbamate (7). Compound **7** was prepared analogous to the procedure of **6** by using 5-amino-1-cyclopentyl-1*H*-pyrazole-4-carboxamide **5c** and (R)-ethyl-2-(((benzyloxy)carbonyl)amino)propanoate **2b** as starting material (yield = 29 %). ^{1}H NMR (400 MHz, $CDCl_3$) δ 11.90 (s, 1H), 8.05 (s, 1H), 7.32 – 7.28 (m, 5H), 5.98 (d, *J* = 6.1 Hz, 1H), 5.23 – 5.05 (m, 3H), 4.98 – 4.83 (m, 1H), 2.10 (dd, *J* = 13.6, 6.9 Hz, 4H), 2.02 – 1.92 (m, 2H), 1.79 – 1.69 (m, 2H), 1.60 (d, *J* = 7.1 Hz, 3H). ^{13}C NMR (101 MHz, $CDCl_3$) δ 160.26, 159.79, 155.83, 151.90, 136.12, 134.56, 128.50, 128.18 (d, *J* = 7.5 Hz), 104.41, 67.18, 57.89, 50.43, 32.47, 24.78, 20.56.

(R)-benzyl-(1-(4-oxo-1-(tetrahydro-2*H*-pyran-4-yl)-4,5-dihydro-1*H*-pyrazolo[3,4-d]pyrimidin-6-yl)ethyl)carbamate (8). Compound **8** was prepared analogous to the procedure of **6** by using 5-amino-1-(tetrahydro-2*H*-pyran-4-yl)-1*H*-pyrazole-4-carboxamide **5d** and (R)-ethyl 2-(((benzyloxy)carbonyl)amino)propanoate **2b** as starting material (yield = 28 %). ^{1}H NMR (400

MHz, $CDCl_3$) δ 12.00 (s, 1H), 8.06 (s, 1H), 7.31 (s, 4H), 7.19 (s, 1H), 5.98 (d, *J* = 7.0 Hz, 1H), 5.11 (dd, *J* = 31.5, 12.2 Hz, 2H), 4.88 (dd, *J* = 23.2, 9.3 Hz, 2H), 4.14 (dd, *J* = 11.3, 2.7 Hz, 2H), 3.61 (t, *J* = 11.5 Hz, 2H), 2.46 – 2.28 (m, 2H), 1.91 (d, *J* = 11.0 Hz, 2H), 1.61 (d, *J* = 7.1 Hz, 3H). ^{13}C NMR (101 MHz, $CDCl_3$) δ 160.05, 155.89, 155.87, 151.73, 136.02, 134.75, 128.54, 128.30, 128.18, 104.66, 67.28, 67.02, 53.87, 50.54, 32.19, 20.40.

(S)-6-(1-aminoethyl)-1-cyclopentyl-1*H*-pyrazolo[3,4-d]pyrimidin-4(5*H*)-one (9). Compound **6** (0.67 g, 1.7 mmol) was dissolved in 20 mL of MeOH and then treated by Pd / C (0.12 g, 10 %). The reaction mixture was stirred under H_2 atmosphere for 12 h. After Pd / C was filtered off, the filtrate was concentrated under vacuum, and the residue was purified by flash column chromatography (dichloromethane / methanol = 80 / 1) to give product as white solid (0.34 g, yield = 81 %). ^{1}H NMR (400 MHz, $CDCl_3$) δ 8.05 (s, 1H), 5.22 – 5.08 (m, 1H), 4.12 (q, *J* = 6.8 Hz, 1H), 2.20 – 2.03 (m, 4H), 2.01 – 1.91 (m, 2H), 1.76 – 1.67 (m, 2H), 1.53 (d, *J* = 6.9 Hz, 3H). ^{13}C NMR (101 MHz, $CDCl_3$) δ 162.65, 158.53, 152.48, 134.49, 104.72, 57.81, 49.70, 32.44, 32.32, 24.75, 23.15.

(R)-6-(1-aminoethyl)-1-cyclopentyl-1*H*-pyrazolo[3,4-d]pyrimidin-4(5*H*)-one (10). Prepared according to the same procedure as **9** by using compound **7** as the starting material (yield = 80%) ^{1}H NMR (400 MHz, $CDCl_3$) δ 8.05 (s, 1H), 5.22 – 5.08 (m, 1H), 4.12 (q, *J* = 6.8 Hz, 1H), 2.20 – 2.03 (m, 4H), 2.01 – 1.91 (m, 2H), 1.76 – 1.67 (m, 2H), 1.53 (d, *J* = 6.9 Hz, 3H). ^{13}C NMR (101 MHz, $CDCl_3$) δ 162.65, 158.53, 152.48, 134.49, 104.72, 57.81, 49.70, 32.44, 32.32, 24.75, 23.15.

(R)-6-(1-aminoethyl)-1-(tetrahydro-2*H*-pyran-4-yl)-1*H*-pyrazolo[3,4-d]pyrimidin-4(5*H*)-one (11). Prepared according to the same procedure as **9** by using compound **8** as the starting material (yield = 83%). ^{1}H NMR (400 MHz, $CDCl_3$) δ 8.05 (s, 1H), 4.86 – 4.78 (m, 1H), 4.15 – 4.09 (m, 3H),

3.63 – 3.56 (m, 2H), 2.44 – 2.31 (m, 2H), 1.93 – 1.87 (m, 2H), 1.52 (d, *J* = 6.9 Hz, 3H). ^{13}C NMR (101 MHz, $CDCl_3$) δ 163.11, 158.69, 152.25, 134.61, 104.85, 67.01, 53.80, 49.80, 32.17, 32.11.

(R)-1-cyclopentyl-6-(1-((2-hydroxybenzylidene)amino)ethyl)-1*H*-pyrazolo[3,4-d]pyrimidin-4(5*H*)-one (12). (R)-6-(1-aminoethyl)-1-cyclopentyl-1*H*-pyrazolo[3,4-d]pyrimidin-4(5*H*)-one **10** was dissolved (0.124 g, 0.5 mmol) in 5 mL of MeOH. Then, salicyaldehyde (0.061 g, 0.5 mmol) was added and the reaction solution was stirred at ambient temperature for 10 h. The solid was filtered to give compound **12** as yellow solid (0.131 g, yield = 69%). M p. = 204.6 – 205.8 ℃. ^{1}H NMR (400 MHz, $CDCl_3$) δ 12.30 (s, 1H), 11.41 (s, 1H), 8.60 (s, 1H), 8.01 (s, 1H), 7.35 – 7.29 (m, 2H), 6.96 – 6.84 (m, 2H), 5.27 – 5.16 (m, 1H), 4.60 (q, *J* = 6.7 Hz, 1H), 2.24 – 2.08 (m, 4H), 2.04 – 1.93 (m, 2H), 1.77 (d, *J* = 6.8 Hz, 3H), 1.76 – 1.71 (m, 2H). ^{13}C NMR (101 MHz, $CDCl_3$) δ 167.27, 160.65, 159.54, 158.79, 151.87, 134.65, 133.20, 132.08, 119.03, 118.57, 117.23, 104.71, 68.03, 58.03, 32.49, 32.41, 24.80, 21.54. HRMS m/z $[M+H]^+$ calcd for: 352.1774, found: 352.1765. HPLC purity = 97.5%.

(R)-1-cyclopentyl-6-(1-((2-hydroxy-4-methoxybenzylidene)amino)ethyl)-1*H*-pyrazolo[3,4-d]pyrimidin-4(5*H*)-one (13). Compound **13** was prepared in a procedure analogous to **12** by using 2-hydroxy-4-methoxybenzaldehyde and **10** as starting materials as white solid (yield = 65 %). M p. = 205.8 – 207.3 ℃. ^{1}H NMR (400 MHz, $CDCl_3$) δ 12.64 (s, 1H), 11.02 (s, 1H), 8.48 (s, 1H), 8.04 (s, 1H), 7.18 (d, *J* = 8.3 Hz, 1H), 6.47 – 6.43 (m, 2H), 5.25 – 5.17 (m, 1H), 4.54 (q, *J* = 6.7 Hz, 1H), 3.81 (s, 3H), 2.20 – 2.08 (m, 4H), 2.04 – 1.94 (m, 2H), 1.77 – 1.72 (m, 2H), 1.74 (d, *J* = 6.8 Hz, 3H). ^{13}C NMR (101 MHz, $CDCl_3$) δ 166.16, 163.83, 163.16, 159.80, 159.17, 151.96, 134.63, 133.26, 112.44, 106.92, 104.70, 101.09, 67.63, 58.01, 55.42, 32.48, 32.41, 24.81, 21.51. HRMS m/z $[M+H]^+$ calcd for: 382.1874, found: 382.1869. HPLC purity = 96.5%.

(R)-1-cyclopentyl-6-(1-((2-hydroxy-4-methoxybenzylidene)amino)ethyl)-1*H*-pyrazolo[3,4-d]pyrimidin-4(5*H*)-one (14). Compound **14** was prepared in a procedure analogous to **12** by using 2-hydroxy-4-methoxybenzaldehyde and **11** as starting materials as white solid (yield = 68%). M p. = 207.1 – 208.2 ℃. ^{1}H NMR (400 MHz, $CDCl_3$) δ 12.61 (s, 1H), 11.26 (s, 1H), 8.48 (s, 1H), 8.05 (s, 1H), 7.18 (d, *J* = 8.5 Hz, 1H), 6.49 – 6.41 (m, 2H), 4.92 – 4.84 (m, 1H), 4.54 (q, *J* = 6.7 Hz, 1H), 4.18 – 4.14 (m, 2H), 3.80 (s, 3H), 3.67 – 3.59 (m, 2H), 2.47 – 2.34 (m, 2H), 2.00 – 1.91 (m, 2H), 1.75 (d, *J* = 6.8 Hz, 3H). ^{13}C NMR (101 MHz, $CDCl_3$) δ 166.13, 163.85, 163.08, 159.82, 159.55, 151.79, 134.84, 133.27, 112.45, 106.91, 104.90, 101.11, 67.73, 67.07, 55.43, 54.01, 32.23, 32.21, 21.54. HRMS m/z $[M+H]^+$ calcd for: 398.1823, found: 398.1820. HPLC purity = 98.4%.

(R)-1-cyclopentyl-6-(1-((2-hydroxybenzyl)amino)ethyl)-1*H*-pyrazolo[3,4-d]pyrimidin-4(5*H*)-one (15). A stirred solution of **12** (0.105 g, 0.3 mmol) in 3 mL of MeOH was treated with $NaBH_4$ (0.023 g, 0.6 mmol) at 0 ℃ and then the reaction mixture was stirred in room temperature for 2 h. The reaction was quenched with 5 % HCl to adjust pH value to 8 ~ 9, and then extracted by ethyl acetate (15 mL × 3). The combined organic phase was concentrated under vacuum to give crude the product, which was purified by flash column chromatography (dichloromethane / methanol = 100 / 1) to afford compound **15** as white solid (94 mg, yield = 89%). M p. = 155.0 – 156.6 ℃. ^{1}H NMR (400 MHz, $CDCl_3$) δ 12.27 (s, 1H), 8.02 (s, 1H), 7.15 (t, *J* = 7.3 Hz, 1H), 6.94 – 6.89 (m, 2H), 6.73 (t, *J* = 7.1 Hz, 1H), 5.29 – 5.15 (m, 1H), 4.02 – 3.97 (m, 2H), 3.81 (d, *J* = 13.2 Hz, 1H), 2.18 – 2.05 (m, 4H), 2.02 – 1.95 (m, 2H), 1.75 – 1.69 (m, 2H), 1.57 (d, *J* = 6.6 Hz, 3H). ^{13}C NMR (101 MHz, $CDCl_3$) δ 161.21, 160.35, 157.40, 152.25, 134.49, 129.08, 128.93, 122.87, 119.31, 116.40, 104.55, 58.05,

56.10, 50.33, 32.42, 32.41, 24.75, 21.33. HRMS m/z $[M+H]^+$ calcd for: 354.1925, found: 354.1925. HPLC purity = 99.1%.

(R)-1-cyclopentyl-6-(1-((2-hydroxy-4-methoxybenzyl)amino)ethyl)-1*H*-pyrazolo[3,4-d]pyrimidin-4(5*H*)-one (16). Compound **16** was prepared in a procedure analogous to **15** using **13** as starting materials as white solid (yield = 78%). M p. =184.4 – 186.1 ℃. ^{1}H NMR (400 MHz, $CDCl_3$) δ 8.04 (s, 1H), 6.84 (d, *J* = 8.3 Hz, 1H), 6.48 (d, *J* = 2.5 Hz, 1H), 6.31 (dd, *J* = 8.3, 2.5 Hz, 1H), 5.26 – 5.17 (m, 1H), 3.96 – 3.91 (m, 2H), 3.78 – 3.71 (m, 4H), 2.19 – 2.07 (m, 4H), 2.02 – 1.94 (m, 2H), 1.76 – 1.72 (m, 2H), 1.55 (d, *J* = 6.9 Hz, 3H). ^{13}C NMR (101 MHz, $CDCl_3$) δ 161.19, 160.66, 160.22, 158.49, 152.21, 134.47, 129.43, 115.20, 105.08, 104.53, 102.15, 58.02, 55.91, 55.24, 49.79, 32.42, 32.40, 29.68, 24.75, 21.28. HRMS m/z $[M+H]^+$ calcd for: 384.2030, found: 384.2031. HPLC purity = 97.9%.

(R)-6-(1-((2-hydroxy-4-methoxybenzyl)amino)ethyl)-1-(tetrahydro-2*H*-pyran-4-yl)-1*H*-pyrazolo[3,4-d]pyrimidin-4(5*H*)-one (17)

Compound **17** was prepared in a procedure analogous to **15** using **14** as starting materials as white solid (yield = 87%). M p. = 179.6 – 180.6 ℃. ^{1}H NMR (400 MHz, $CDCl_3$) δ 8.05 (s, 1H), 6.84 (d, *J* = 8.3 Hz, 1H), 6.49 (d, *J* = 2.5 Hz, 1H), 6.30 (dd, *J* = 8.3, 2.5 Hz, 1H), 4.97 – 4.89 (m, 1H), 4.22 – 4.09 (m, 2H), 3.98 (q, *J* = 6.8 Hz, 1H), 3.93 – 3.89 (m, 1H), 3.78 – 3.74 (m, 1H), 3.76 (s, 3H), 3.70 – 3.60 (m, 2H), 2.45 – 2.32 (m, 2H), 1.99 – 1.90 (m, 2H), 1.56 (d, *J* = 6.8 Hz, 3H). ^{13}C NMR (101 MHz, $CDCl_3$) δ 161.62, 160.74, 160.17, 158.39, 152.04, 134.68, 129.45, 115.33, 105.05, 104.74, 102.18, 67.02, 55.99, 55.27, 53.91, 49.76, 32.30, 32.17, 21.33. HRMS m/z $[M+H]^+$ calcd for: 400.1979, found: 400.1979. HPLC purity = 97.6%.

(S)-1-cyclopentyl-6-(1-((2-hydroxybenzyl)amino)ethyl)-1*H*-pyrazolo[3,4-d]pyrimidin-4(5*H*)-one (18). (S)-6-(1-aminoethyl)-1-cyclopentyl-1*H*-pyrazolo[3,4-d]pyrimidin-4(5*H*)-one (**9**) was dissolved (0.124 g, 0.5 mmol) in 5 mL of MeOH. Salicyaldehyde (0.061 g, 0.5 mmol) was added and the reaction solution was stirred for 2 h at room temperature. $NaBH_4$ (0.038 g, 1.0 mmol) was added to the reaction slowly at 0 ℃ and the mixture was stirred in room temperature for additional 2 h. The reaction was quenched with 5 % HCl to adjusted pH value to 8 ~ 9 and extracted with ethyl acetate (15 mL×3). The combined organic layer was concentrated under vacuum to give crude product, the following purification by flash column chromatography (dichloromethane / methanol = 100 / 1) afford **18** as white solid (158 mg, yield = 90%). M p. = 154.6 – 155.7 ℃. ^1H NMR (400 MHz, $CDCl_3$) δ 12.27 (s, 1H), 8.02 (s, 1H), 7.15 (t, *J* = 7.3 Hz, 1H), 6.94 – 6.89 (m, 2H), 6.73 (t, *J* = 7.1 Hz, 1H), 5.29 – 5.15 (m, 1H), 4.02 - 3.97 (m, 2H), 3.81 (d, *J* = 13.2 Hz, 1H), 2.18 – 2.05 (m, 4H), 2.02 – 1.95 (m, 2H), 1.75 – 1.69 (m, 2H), 1.57 (d, *J* = 6.6 Hz, 3H). ^{13}C NMR (101 MHz, $CDCl_3$) δ 161.21, 160.35, 157.40, 152.25, 134.49, 129.08, 128.93, 122.87, 119.31, 116.40, 104.55, 58.05, 56.10, 50.33, 32.42, 32.41, 24.75, 21.33. HRMS m/z $[M+H]^+$ calcd for: 354.1925, found: 354.1918. HPLC purity = 98.1%.

(R)-1-cyclopentyl-6-(1-((2-hydroxy-5-methoxybenzyl)amino)ethyl)-1*H*-pyrazolo[3,4-d]pyrimidin-4(5*H*)-one (19). Compound **19** was prepared in a procedure analogous to **18** using **10** and 2-hydroxy-5-methoxybenzaldehyde as starting materials as white solid (yield = 76 %). M p. = 64.2 – 66.0 ℃. ^1H NMR (400 MHz, $CDCl_3$) δ 8.02 (s, 1H), 6.83 (d, *J* = 8.8 Hz, 1H), 6.70 (dd, *J* = 8.8, 3.0 Hz, 1H), 6.52 (d, *J* = 3.0 Hz, 1H), 5.27 – 5.15 (m, 1H), 4.01 – 3.90 (m, 2H), 3.76 (d, *J* = 13.2 Hz, 1H), 3.67 (s, 3H), 2.18 – 2.07 (m, 4H), 2.03 – 1.93 (m, 2H), 1.79 – 1.73 (m, 2H), 1.56 (d, *J* = 6.9 Hz,

3H). ^{13}C NMR (101 MHz, $CDCl_3$) δ 161.30, 160.34, 152.64, 152.25, 151.11, 134.50, 123.62, 116.75, 114.97, 113.77, 104.54, 58.03, 56.11, 55.68, 50.37, 32.43, 32.39, 24.74, 21.30. HRMS m/z $[M+H]^+$ calcd for: 384.2030, found: 384.2032. HPLC purity = 97.4%.

(R)-1-cyclopentyl-6-(1-((2-hydroxy-3-methoxybenzyl)amino)ethyl)-1*H*-pyrazolo[3,4-d]pyrimidin-4(5*H*)-one (20). Compound **20** was prepared in a procedure analogous to **18** using **10** and 2-hydroxy-3-methoxybenzaldehyde as starting materials as white solid (yield = 76 %). M p. = 129.8 – 131.6 ℃. ^{1}H NMR (400 MHz, $CDCl_3$) δ 8.05 (s, 1H), 6.82 (dd, *J* = 7.9, 1.6 Hz, 1H), 6.78 (t, *J* = 7.7 Hz, 1H), 6.72 (dd, *J* = 7.3, 1.5 Hz, 1H), 5.21 – 5.13 (m, 1H), 3.88 (s, 3H), 3.87 – 2.83 (m, 2H), 3.72 (d, *J* = 12.7 Hz, 1H), 2.14 – 2.05 (m, 4H), 2.02 – 1.94 (m, 2H), 1.78 – 1.72 (m, 2H), 1.45 (d, *J* = 6.8 Hz, 3H). ^{13}C NMR (101 MHz, $CDCl_3$) δ 161.81, 158.96, 152.47, 147.17, 145.10, 134.54, 124.06, 121.81, 119.34, 110.77, 104.93, 57.85, 56.15, 56.07, 48.72, 32.42, 32.36, 24.74, 21.59. HRMS m/z $[M+H]^+$ calcd for: 384.2030, found: 384.2031. HPLC purity = 98.6%.

(R)-6-(1-((5-chloro-2-hydroxybenzyl)amino)ethyl)-1-cyclopentyl-1*H*-pyrazolo[3,4-d]pyrimidin-4(5*H*)-one (21). Compound **21** was prepared in a procedure analogous to **18** using **10** and 5-chloro-2-hydroxybenzaldehyde as starting materials as white solid (yield = 77 %). M p = 172.4 – 173. 6 ℃. ^{1}H NMR (400 MHz, $CDCl_3$) δ 12.36 (s, 1H), 8.01 (s, 1H), 7.08 – 7.06 (m, 1H), 6.89 (d, *J* = 2.3 Hz, 1H), 6.84 (d, *J* = 8.6 Hz, 1H), 5.28 – 5.15 (m, 1H), 4.06 – 3.92 (m, 2H), 3.74 (d, *J* = 13.5 Hz, 1H), 2.22 – 2.08 (m, 4H), 2.04 – 1.94 (m, 2H), 1.81 – 1.70 (m, 2H), 1.58 (d, *J* = 6.8 Hz, 3H). ^{13}C NMR (101 MHz, $CDCl_3$) δ 160.86, 160.44, 156.13, 152.18, 134.47, 128.80, 128.58, 124.13, 123.78, 117.68, 104.46, 58.07, 55.93, 49.89, 32.45, 32.42, 24.74, 21.24. HRMS m/z $[M+H]^+$ calcd for: 388.1535, found: 388.1525. HPLC purity = 99.1%.

(R)-1-cyclopentyl-6-(1-((5-fluoro-2-hydroxybenzyl)amino)ethyl)-1*H*-pyrazolo[3,4-d]pyrimidin-4(5*H*)-one (22). Compound **22** was prepared in a procedure analogous to **18** using **10** and 5-fluoro-2-hydroxybenzaldehyde as starting materials as white solid (yield = 84%). M p = 157.4 – 158.9 ℃. ^{1}H NMR (400 MHz, $CDCl_3$) δ 8.02 (s, 1H), 6.83 – 6.82 (m, 2H), 6.65 (d, *J* = 8.5 Hz, 1H), 5.27 – 5.16 (m, 1H), 4.02 – 3.89 (m, 2H), 3.76 (d, *J* = 13.4 Hz, 1H), 2.19 – 2.07 (m, 4H), 2.03 – 1.93 (m, 2H), 1.80 – 1.72 (m, 2H), 1.57 (d, *J* = 6.9 Hz, 3H). ^{13}C NMR (101 MHz, $CDCl_3$) δ 161.02, 160.41, 157.21, 154.86, 153.33, 153.31, 152.20, 134.44, 123.77, 123.70, 117.05, 116.97, 115.43, 115.23, 115.20, 115.00, 104.50, 58.09, 56.06, 50.01, 32.43, 32.41, 24.75, 21.25. HRMS m/z $[M+H]^+$ calcd for: 372.1830, found: 372.1822. HPLC purity = 98.6%.

(R)-1-cyclopentyl-6-(1-((2-hydroxy-5-nitrobenzyl)amino)ethyl)-1*H*-pyrazolo[3,4-d]pyrimidin-4(5*H*)-one (23). Compound **23** was prepared in a procedure analogous to **18** using **10** and 2-hydroxy-5-nitrobenzaldehyde as starting materials as yellow solid (yield = 69 %). ^{1}H NMR (400 MHz, $CDCl_3$) δ 8.03 (dd, *J* = 9.0, 2.7 Hz, 1H), 7.96 (s, 1H), 7.89 (d, *J* = 2.6 Hz, 1H), 6.96 (d, *J* = 9.0 Hz, 1H), 5.27 – 5.17 (m, 1H), 4.11 (d, *J* = 13.9 Hz, 1H), 3.99 (q, *J* = 6.8 Hz, 1H), 3.88 (d, *J* = 13.9 Hz, 1H), 2.21 – 2.09 (m, 4H), 2.03 – 1.95 (m, 2H), 1.81 – 1.70 (m, 2H), 1.60 (d, *J* = 6.8 Hz, 3H). ^{13}C NMR (101 MHz, $CDCl_3$) δ 163.96, 160.43, 152.10, 140.20, 134.35, 125.55, 125.08, 122.73, 116.70, 104.32, 58.16, 55.63, 49.72, 32.47, 32.44, 24.77, 21.16. HRMS m/z $[M+H]^+$ calcd for: 399.1775, found: 399.1779. HPLC purity = 99.2%.

(R)-1-cyclopentyl-6-(1-((5-(dimethylamino)-2-hydroxybenzyl)amino)ethyl)-1*H*-pyrazolo[3,4-d] pyrimidin-4(5*H*)-one (24). Compound **24** was prepared in a procedure analogous to **18** using **10** and 5-(dimethylamino)-2-hydroxybenzaldehyde as starting materials as brown solid (yield = 64%). ^{1}H

NMR (400 MHz, $CDCl_3$) δ 8.03 (s, 1H), 6.82 (d, *J* = 8.2 Hz, 1H), 6.64 (d, *J* = 7.1 Hz, 1H), 6.43 (s, 1H), 5.27 – 5.16 (m, 1H), 4.01 – 3.93 (m, 2H), 3.77 (d, *J* = 12.8 Hz, 1H), 2.76 (s, 6H), 2.19 – 2.06 (m, 4H), 2.03 – 1.92 (m, 2H), 1.79 – 1.67 (m, 2H), 1.55 (d, *J* = 6.8 Hz, 3H). ^{13}C NMR (101 MHz, $CDCl_3$) δ 161.43, 160.22, 152.27, 149.33, 144.77, 134.55, 123.31, 116.78, 115.17, 114.84, 104.57, 57.97, 56.11, 50.77, 41.96, 32.44, 32.38, 24.73, 21.37. HRMS m/z $[M+H]^+$ calcd for: 397.2347, found: 397.2338. HPLC purity = 97.8%.

(S)-1-cyclopentyl-6-(1-((pyridin-2-ylmethyl)amino)ethyl)-1*H*-pyrazolo[3,4-d]pyrimidin-4(5*H*)-one (25). Compound **25** was prepared in a procedure analogous to **18** by using **9** and 2-pyridinecarboxaldehyde as starting materials as white solid (yield = 59%). M p. = 106.0 – 107.9 ℃. ^1H NMR (400 MHz, $CDCl_3$) δ 8.59 (d, *J* = 4.6 Hz, 1H), 8.05 (s, 1H), 7.67 – 7.62 (m, 1H), 7.20 (t, *J* = 6.4 Hz, 2H), 5.21 – 5.07 (m, 1H), 3.94 – 3.77 (m, 3H), 2.18 – 2.05 (m, 4H), 2.02 – 1.95 (m, 2H), 1.75 - 1.69 (m, 2H), 1.50 (d, *J* = 6.8 Hz, 3H). ^{13}C NMR (101 MHz, $CDCl_3$) δ 161.90, 158.35, 158.09, 152.43, 149.36, 136.68, 134.52, 122.39, 105.02, 57.78, 56.90, 53.15, 32.41, 32.35, 24.72, 21.46. HRMS m/z $[M+H]^+$ calcd for: 339.1928, found: 339.1918. HPLC purity = 96.3 %.

(R)-1-cyclopentyl-6-(1-((pyridin-2-ylmethyl)amino)ethyl)-1*H*-pyrazolo[3,4-d]pyrimidin-4(5*H*)-one (26). Compound **26** was prepared in a procedure analogous to **18** by using **10** and 2-pyridinecarboxaldehyde as starting materials as white solid (yield = 59%). M p. = 105.3 – 106.7 ℃. ^1H NMR (400 MHz, $CDCl_3$) δ 8.59 (d, *J* = 4.6 Hz, 1H), 8.05 (s, 1H), 7.67 – 7.62 (m, 1H), 7.20 (t, *J* = 6.3 Hz, 2H), 5.21 – 5.07 (m, 1H), 3.94 – 3.79 (m, 3H), 2.30 (s, 1H), 2.16 – 2.05 (m, 4H), 2.01 – 1.91 (m, 2H), 1.77 – 1.66 (m, 2H), 1.52 (d, *J* = 6.8 Hz, 3H). ^{13}C NMR (101 MHz, $CDCl_3$) δ 161.88, 158.25, 158.03, 152.44, 149.38, 136.68, 134.53, 122.41, 122.38, 105.04, 57.75, 56.88, 53.16,

32.42, 32.36, 24.72, 21.51. HRMS m/z $[M+H]^+$ calcd for: 339.1928, found: 339.1924. HPLC purity = 97.2 %.

(R)-1-cyclopentyl-6-(1-(((6-methylpyridin-2-yl)methyl)amino)ethyl)-1H-pyrazolo[3,4-d]pyrimidin-4(5H)-one (27). Compound **27** was prepared in a procedure analogous to **18** using **10** and 6-methyl-2-pyridinecarboxaldehyde as starting materials as white solid (yield = 59 %). M p. = 103.4 – 105.1 ℃. ^{1}H NMR (400 MHz, $CDCl_3$) δ 8.03 (s, 1H), 7.51 (t, *J* = 7.6 Hz, 1H), 7.01 (dd, *J* = 17.8, 7.6 Hz, 2H), 5.21 – 5.06 (m, 1H), 3.89 – 3.76 (m, 3H), 2.55 (s, 3H), 2.16 – 2.04 (m, 4H), 1.99 – 1.94 (m, 2H), 1.75 – 1.65 (m, 2H), 1.50 (d, *J* = 6.8 Hz, 3H). ^{13}C NMR (101 MHz, $CDCl_3$) δ 162.00, 158.40, 158.22, 157.28, 152.45, 136.89, 134.52, 121.96, 119.31, 105.04, 57.73, 56.86, 53.24, 32.42, 32.35, 24.72, 24.33, 21.51. HRMS m/z $[M+H]^+$ calcd for: 353.2084, found: 353.2077. HPLC purity = 98.0%.

(R)-6-(1-(((6-bromopyridin-2-yl)methylene)amino)ethyl)-1-cyclopentyl-1H-pyrazolo[3,4-d]pyrimidin-4(5H)-one (28). Compound **28** was prepared in a procedure analogous to **12** using **10** and 6-bromoypyridine-2-carbaldehyde as starting materials as yellow solid (yield = 81%). M p. = 171.8 – 173.4 ℃. ^{1}H NMR (400 MHz, $CDCl_3$) δ 7.99 (s, 1H), 7.57 (t, *J* = 7.7 Hz, 1H), 7.50 – 7.48 (m, 1H), 7.45 -7.43 (m, 1H), 6.33 (d, *J* = 5.7 Hz, 1H), 5.24 – 5.16 (m, 1H), 4.87 – 4.78 (m, 1H), 3.00 (t, *J* = 7.4 Hz, 1H), 2.18 – 2.07 (m, 4H), 2.01 – 1.97 (m, 2H), 1.77 – 1.71 (m, 2H), δ 1.60 (d, *J* = 6.9 Hz, 3H). ^{13}C NMR (101 MHz, $CDCl_3$) δ 163.22, 157.71, 155.81, 153.25, 142.19, 139.10, 134.50, 128.52, 121.91, 104.65, 74.90, 57.93, 56.69, 32.51, 32.37, 24.78, 18.50. HRMS m/z $[M+Na]^+$ calcd for: 437.0696, found: 437.0691. HPLC purity = 96.5%.

(R)-6-(1-(((6-bromopyridin-2-yl)methyl)amino)ethyl)-1-cyclopentyl-1H-pyrazolo[3,4-d]pyrimidin-4(5H)-one (29). Compound **29** was prepared in a procedure analogous to **15** by using **28** as starting materials as white solid (yield = 69%). M p. = 96.1 – 97.6 ℃. ^{1}H NMR (400 MHz, $CDCl_3$) δ 10.49 (s, 1H), 8.05 (s, 1H), 7.51 (t, *J* = 7.7 Hz, 1H), 7.38 (d, *J* = 7.8 Hz, 1H), 7.21 (d, *J* = 7.4 Hz, 1H), 5.23 – 5.09 (m, 1H), 3.92 – 3.75 (m, 3H), 2.47 (s, 1H), 2.18 – 2.04 (m, 4H), 2.01 – 1.92 (m, 2H), 1.77 – 1.66 (m, 2H), 1.52 (d, *J* = 6.8 Hz, 3H). ^{13}C NMR (101 MHz, $CDCl_3$) δ 161.61, 159.79, 158.30, 152.36, 141.97, 138.99, 134.54, 126.78, 121.19, 104.97, 57.78, 56.83, 52.63, 32.44, 32.37, 24.72, 21.58. HRMS m/z $[M+H]^+$ calcd for: 417.1033, found: 417.1028. HPLC purity = 98.9%.

Reference

(1) Di, L.; Kerns, E. H.; Fan, K.; McConnell, O. J.; Carter, G. T., High throughput artificial membrane permeability assay for blood–brain barrier. *Eur. J. Med. Chem.* **2003**, *38*, 223-232.
